# Co-embedding of edges and nodes with deep graph convolutional neural networks

**DOI:** 10.1038/s41598-023-44224-1

**Published:** 2023-10-08

**Authors:** Yuchen Zhou, Hongtao Huo, Zhiwen Hou, Lingbin Bu, Jingyi Mao, Yifan Wang, Xiaojun Lv, Fanliang Bu

**Affiliations:** 1 People’s Public Security University of China, Beijing, 100038 China; 2 China Academy of Railway Sciences Corporation Limited, Beijing, 100081 China

**Keywords:** Mathematics and computing, Computer science

## Abstract

Graph neural networks (GNNs) have significant advantages in dealing with non-Euclidean data and have been widely used in various fields. However, most of the existing GNN models face two main challenges: (1) Most GNN models built upon the message-passing framework exhibit a shallow structure, which hampers their ability to efficiently transmit information between distant nodes. To address this, we aim to propose a novel message-passing framework, enabling the construction of GNN models with deep architectures akin to convolutional neural networks (CNNs), potentially comprising dozens or even hundreds of layers. (2) Existing models often approach the learning of edge and node features as separate tasks. To overcome this limitation, we aspire to develop a deep graph convolutional neural network learning framework capable of simultaneously acquiring edge embeddings and node embeddings. By utilizing the learned multi-dimensional edge feature matrix, we construct multi-channel filters to more effectively capture accurate node features. To address these challenges, we propose the Co-embedding of Edges and Nodes with Deep Graph Convolutional Neural Networks (CEN-DGCNN). In our approach, we propose a novel message-passing framework that can fully integrate and utilize both node features and multi-dimensional edge features. Based on this framework, we develop a deep graph convolutional neural network model that prevents over-smoothing and obtains node non-local structural features and refined high-order node features by extracting long-distance dependencies between nodes and utilizing multi-dimensional edge features. Moreover, we propose a novel graph convolutional layer that can learn node embeddings and multi-dimensional edge embeddings simultaneously. The layer updates multi-dimensional edge embeddings across layers based on node features and an attention mechanism, which enables efficient utilization and fusion of both node and edge features. Additionally, we propose a multi-dimensional edge feature encoding method based on directed edges, and use the resulting multi-dimensional edge feature matrix to construct a multi-channel filter to filter the node information. Lastly, extensive experiments show that CEN-DGCNN outperforms a large number of graph neural network baseline methods, demonstrating the effectiveness of our proposed method.

## Introduction

**Figure 1:**
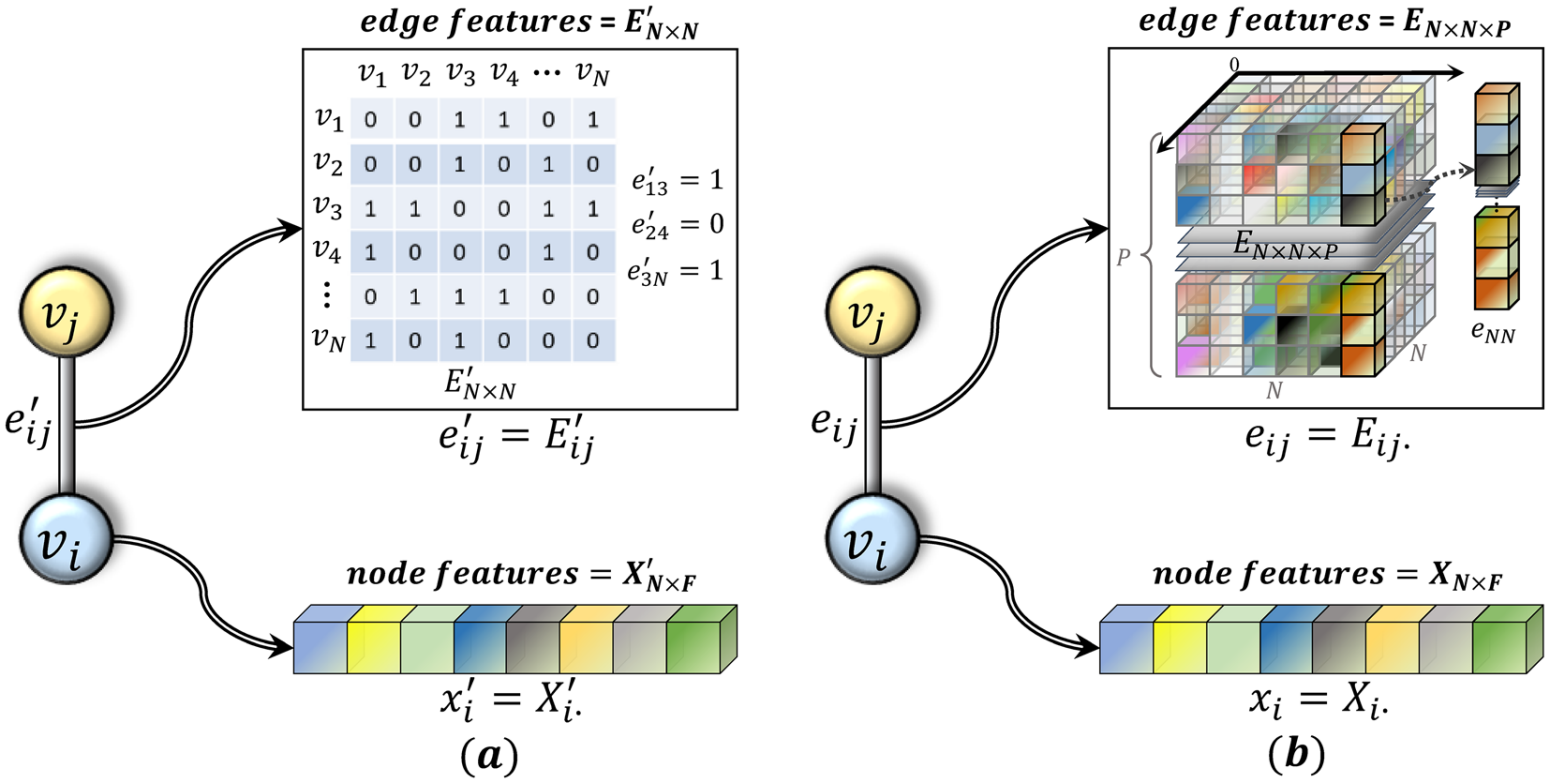
(**a**) The edge feature representation and node feature representation in ordinary GCN; (**b**) The edge feature representation and node feature representation used in our proposed CEN-DGCNN.

Graphs usually contain rich node features and edge features. However, in recent years, the majority of advanced GNN models have primarily focused on enhancing the learning of node features, while ignoring the synchronous learning of edge features. Although the aggregation function designed based on the message passing neural network framework (MPNN)^1^ can aggregate node features and edge features, and achieve good results in specific application scenarios. But using predefined aggregation functions is more like manual feature engineering and cannot be applied in all cases. Therefore, we hope to achieve a method that can learn multi-dimensional edge features iteratively, and synchronize the update of multi-dimensional edge features to the process of node information aggregation, giving full play to the role of node features and edge features. The edge features in the conventional graph convolution neural networks (GCNs) shown in Fig. 1a are represented by the adjacency matrix, which can only be represented by binary indicator variables or one-dimensional real values, and cannot express rich edge information. Figure 1b shows the multi-dimensional edge feature representation proposed by us. The edge feature is no longer represented by a one-dimensional real value in a simple adjacency matrix, but is represented by a learnable feature vector, which can express rich edge information and can be updated across layers.

Explanation of the symbols in Fig. 1: Assuming we have a graph $G$ consisting of $N$ nodes, where ${v}_{i}$ and ${v}_{j}$ represent nodes $i$ and $j$, ${e}_{ij}{\prime}$ and ${e}_{ij}$ correspond to the edge feature representations of edge $ij$ in ordinary GCN and ME-DGCNN, while ${x}_{i}{\prime}$ and ${x}_{i}$ respectively represent the feature vectors of node $i$ in ordinary GCN and ME-DGCNN. ${E}_{N\times N}{\prime}$ and ${E}_{N\times N\times P}$ represent the edge feature matrix (a two-dimensional tensor) of ordinary GCN and the multi-dimensional edge feature matrix (a three-dimensional tensor) of ME-DGCNN, respectively. We use the ∙ notation to indicate selecting the entire range (slicing) along the respective dimensions. Therefore, ${E}_{ij}{\prime}$ (a scalar) and ${E}_{ij\cdot }$ (a feature vector) both denote the features of edge $ij$. ${X}_{N\times F}{\prime}$ and ${X}_{N\times F}$ represent the node feature matrix (a two-dimensional tensor) of ordinary GCN and ME-DGCNN, respectively, where ${X}_{i\cdot }{\prime}$ and ${X}_{i\cdot }$ both denote the feature vectors of node $i$. It can be seen from (a) that the ordinary GCN only uses 1 and 0 to denote the presence or absence of edges, and uses the $N\times N$ adjacency matrix as the node information filter. The CEN-DGCNN proposed in (b) will use the *P*-dimensional feature vector to represent the edge feature, and the $N\times N\times P$ edge feature matrix will be used as the multi-channel filter of node information.

The existing GNN methods mainly focus on how to effectively obtain accurate node features, while ignoring the use of edge information. Although the Message Passing Neural Network (MPNN) framework proposed by Gilmer et al.^1^ allows both edge information and node information to participate in the message passing process, but most of the advanced models still focus on node features and ignore edge features. Kipf et al.^2^ simplified the spectral convolution by approximating the Chebyshev polynomials of the graph Laplace operator, and proposed Graph Convolution Networks (GCNs) based on non-spectral method^3^. GCN simplifies the convolution filter by limiting the receptive field to the 1-hop neighbor of each node, but the process of information aggregation does not take into account the different relationship between the node's 1-hop neighbors and the node itself, nor does it consider the edge features. In many scenes, edges can have different category labels. For example, in social networks, edges can be labeled as friend relationships, family relationships, work relationships, and so on. Therefore, Schlichtkrull et al.^4^ proposed Relational Graph Convolutional Networks (R-GCNs) to generalize GCN to graph data with multiple relationships, which can aggregate information according to the type of edges. Most GCN methods use Laplace operator or adjacency matrix to aggregate node information, without taking into account the different connection weights between different node pairs. Veličković et al.^5^ proposed Graph Attention Networks (GATs) to give weights to different node pairs according to their characteristics, which trains the weight coefficients associated with their neighbors for each node. In essence, the weight in GAT is a function of node features, and the attention weight coefficients between two nodes with connected edges are calculated from the feature vectors of two nodes, so GAT has stronger adaptability to the fusion of node features and structural features, and achieves better results. However, the edge also contains rich information. Consequently, we aspire to develop a GNN model that can concurrently learn both node features and edge features. This model will facilitate the learning of node features based on the knowledge acquired from edge features. Such an approach will enable nodes to acquire more precise and comprehensive information, while also learning edge embeddings to enhance the representation of edge features.

The main reason for the success of most of the existing shallow GCN models is that in some application scenarios, the node features mainly rely on the short-range information of their local neighborhood. For example, in social networks, the friendship is limited to the “small world”^6^, and the receptive field can be extended to local neighborhood nodes of several hops only by stacking several layers of GCN. Stacking more layers may even lead to over-smoothing and over-squeezing^7^, which instead makes the performance of the network drop sharply^8^, the over-smoothing problem also exists in the continuous-time GNNs field^9–12^. One of the drawbacks of GNNs is the fixed aggregation distance, which determines the number of other nodes considered relevant to each node and is determined by the number of layers in the GNN model^13^. When the scale of the network becomes larger, or the node features under the special application background need to consider the remote node dependency, a deeper GCN is needed to expand the receptive field. For example, the prediction of molecular chemical properties may require atomic combinations on opposite sides^14^. Li et al.^15^ use the 56-layer graph convolution neural network they constructed to segment the point cloud data semantically, and achieve better performance than the shallow network. Larger graphs and meshes also need deep GCN to capture remote dependencies between nodes^16,17^. But there are still two problems in training deep GCN. One is the phenomenon of over-smoothing: because of the recursive neighborhood aggregation of the model^7,18^, each node aggregates almost global node information to itself, which will cause the characteristics of all nodes to become indistinguishable. The second is the phenomenon of excessive squeeze: because the network is too deep, many iterations will aggregate the information of a large number of neighborhood nodes into themselves and be over-compressed into fixed-size vectors^19^, which may lead to information distortion and make the performance of the deep GNN network model inferior to that of the shallow model. Rusch et al.^20^ proposed that alleviating over-smoothing is a necessary condition for the construction of deep GNNs. In pursuit of enabling nodes to effectively aggregate information from distant nodes, thus acquiring non-local structural features and more sophisticated node features, our objective is to establish a deep graph neural network framework that fulfills these criteria while mitigating the issues of over-smoothing and over-squeezing.

We propose a co-embedding of edges and nodes with deep graph convolutional neural network (CEN-DGCNN) for addressing the above problems. We abandon the method of using binary indicator variables or one-dimensional real values to represent edge features in conventional GCNs, and introduce multi-dimensional edge embedding representation to make full use of edge information. And a new message passing framework is being proposed to integrate multi-dimensional edge features and node features, allowing full use of node information and edge information. At the same time, in order to meet the application scenarios that need to capture the remote dependencies of nodes, we also construct a message passing framework which introduces the idea of residual connection and dense connection. Based on this framework, a deep graph convolution neural network can be designed to mine remote dependency relationships between nodes. In addition, we also construct a new graph convolutional layer, each layer can learn node features and edge features simultaneously, and can be updated iteratively across layers. Edge learning and node learning are integrated into the same convolution layer, which greatly improves the efficiency of the model and reduces the complexity of the model. The experimental results demonstrate that our proposed method attains state-of-the-art performance in both the node classification and link prediction tasks, particularly for datasets with directed edges. The contributions of this paper are as follows:

1. We propose a new message passing framework that enables the simultaneous aggregation of multi-dimensional edge and node features. By introducing the idea of residual connection and dense connection, the construction of deep graph convolutional neural network model is realized, which is used to capture the long-range dependency and non-local structural features between nodes.
2. We eliminate the limitation that conventional GNNs only use binary variables or one-dimensional real values to represent edge features, and propose a multi-dimensional edge feature representation method. Our approach uses edge embeddings to encode rich edge information, which can be updated iteratively across graph convolution layers.
3. We design a new graph convolutional layer that can process node and edge embeddings in parallel, allowing edge features to be updated based on node features and attention mechanism. Additionally, we use the multi-dimensional edge feature matrix to construct multi-channel filters for filtering node information, while introducing an identity mapping mechanism to prevent over-smoothing.
4. To handle directed graphs with missing edge features, we propose a multi-dimensional edge feature encoding method and multi-channel filter construction method that takes into account the directionality of edges. Our experimental results demonstrate the effectiveness of these methods.

The rest of this paper is organized as follows: Section “Related work” provides a brief overview of related work on deep graph convolution networks and edge learning. Section “The proposed method: CEN-DGCNN” presents the details of our proposed CEN-DGCNN model. Section “Discussion” gives a brief discussion. Section “Experiments” presents the experimental results. Finally, in Section “Conclusion”, we conclude this paper and summarize our contributions.

## Related work

### Deep graph neural networks-related work

In order to capture the long-range dependencies and non-local structural features between nodes, we hope to build a deep GCN model, but when the model is too deep, it usually appears the phenomenon of over-smoothing^7,21,22^ and over-squeezing^19^, and the node representation will become indistinguishable or distorted, resulting in a great degradation of network performance. Many methods have been proposed on how to deepen GCN. The existing research methods are mainly divided into three categories: architecture modification, graph normalization, and random dropping. Next, we will introduce the above methods.

#### Architecture modification

For architecture modification, most of the existing methods mainly introduce the residual connection^23,24^ in convolutional neural network (CNN) into GCN. Li et al.^15^ borrowed from the concept of CNN, applied methods such as residual connection, dense connection, and dilated convolution to the GCN architecture, and successfully trained a GCN with a depth of up to 56 layers, and proved the effectiveness of the model through the point cloud semantic segmentation task. Chen et al.^25^ also borrowed the concept of residual connection, introduced the initial residual into the graph neural network, and established a graph convolutional network model with a depth of 64 layers, and achieved good results. Xu et al.^26^ proposed the Jumping Knowledge Networks, which differ from common neighborhood aggregation networks that aggregate information from the previous layer in each layer. Instead, in the last layer, it uses residual connections to combine the output of each layer.

#### Graph normalization

Similarly, many studies have been carried out around regularization and normalization methods to try to deepen GNN. Zhao et al.^27^ proposed a new normalized layer PairNorm, which can be applied to the middle layer during training to prevent node embedding from being too similar. Experiments on large data sets show that PairNorm is obviously better than the shallow model. Zhou et al.^8^ pointed out that in stacked multi-layer GCN, propagation operations and transformation operations are performed by each layer graph convolution. Previous studies have focused on the study of propagation operations to alleviate the performance degradation of deep GCN models. Through the research on the transformation operation, Zhou et al. found that its contribution to the performance degradation of the deep model is even greater than that of the propagation operation, and proposed a variance control technique called NodeNorm. Li et al.^28^ also found that normalization technology plays an important role in training depth GCN, so they proposed a message normalization layer called MsgNorm. Zhou et al.^29^ clustered nodes into multiple groups and applied Differentiable Group Normalization (DGN) to each node group separately.

#### Random dropping

In machine learning, if the model is too complex, too many parameters, and the number of training samples is too small, it is easy to produce over-fitting phenomenon. Dropout^30^ effectively alleviates the overfitting problem of the model by randomly discarding the hidden units in the neural network with a preset probability, which makes it possible to train a deeper network. The field of GNN is also inspired, and part of the work introduces the idea of Dropout. Rong et al.^31^ proposed DropEdge to eliminate the over-smoothing problem of deep graph convolutional neural networks by randomly deleting a certain number of edges in the graph at each training epoch. Huang et al.^32^ also proposed to train the model by removing nodes (DropNode). Since when a node is deleted, the edges connected to it will also be deleted, so DropNode can be regarded as a special form of DropEdge. The above two approaches can be viewed as data enhancers and message passing reducers.

### Edge-related work

Since much of the real-world data is in a non-Euclidean form, graph representation learning has made tremendous progress in recent years. The current graph representation learning methods can be roughly divided into three categories: matrix factorization, random walk, and graph neural network. The method based on matrix decomposition is computationally expensive, and the method based on random walk is also difficult to apply in large-scale graphs. The graph neural network method can effectively solve the above problems and has been widely used in recent years. Although graph representation learning has achieved success in many fields, but most methods ignore edge information. In order to utilize edge information, the following methods have been successively proposed.

#### Implicit and simple edge information utilization

The standard GCN method aggregates only the first-order neighbor nodes' information, and its neighboring node judgment is based on whether there are edges between them. Typically, edges are represented as either “1” (for a connection) or “0” (for no connection). As such, the edge features are considered as binary indicator variables that only represent whether an edge exists or not. Alternatively, scalars can be used to represent weighted edges, where the adjacency matrix contains values that indicate the strength of the connecting edges.

#### Aggregate information based on different types of edges

In many specific scenarios, edges can be labeled with different types of annotations. For example, in a social network, edges can be marked as friend relationships, colleague relationships, classmate relationships, relative relationships, etc. A common approach is to aggregate information separately based on different edge types. Schlichtkrull et al.^4^ proposed Relational Graph Convolutional Networks (R-GCNs) in order to solve the disadvantages of ordinary GCNs that did not consider the relationship between nodes during the information aggregation process. The specific message passing model is as follows:

1 $${h}_{i}^{\left(l+1\right)}=\sigma \left(\sum_{r\in R}\sum_{j\in {N}_{i}^{r}}\frac{1}{{c}_{i,r}}{W}_{r}^{\left(l\right)}{h}_{j}^{\left(l\right)}+{W}_{0}^{\left(l\right)}{h}_{i}^{\left(l\right)}\right)$$

In the Eq. (1) above, ${h}_{i}^{\left(l+1\right)}$ represents the output features of node $i$ in the (*l* + 1)th layer of the R-GCN, while ${h}_{i}^{\left(l\right)}$ and ${h}_{j}^{\left(l\right)}$ represent the outputs of nodes $i$ and $j$ in the *l*th layer. The $\sigma$ denotes the activation function. In these expressions, $R$ represents the set of all relations,${N}_{i}^{r}$ represents the set of first-order neighbor nodes connected to node $i$ with the relationship category denoted as $r$. ${c}_{i,r}$ is a regularization constant with a value of $|{N}_{i}^{r}|$, and ${W}_{r}^{\left(l\right)}$ denotes the weight parameter matrix employed in the *l*th layer of the model for linear transformation of neighbor nodes with relation category $r$. This matrix is used to transform the features of neighbor nodes connected by edges of the same type. ${W}_{0}^{\left(l\right)}$ represents the weight parameter matrix associated with the node itself in the *l*th layer. In contrast to typical GCNs that aggregate messages from all first-order neighbor nodes uniformly, R-GCNs aggregate messages from various types of first-order neighbor nodes differentially. However, this method only addresses edges with certain types and cannot handle edges with multi-dimensional features.

#### Multi-dimensional edge feature aggregation

The previously mentioned methods cannot effectively utilize and handle multi-dimensional edge features, so some studies are exploring how to use the multi-dimensional edge features. The common method is to aggregate the multi-dimensional edge features and neighbor node features together through the aggregation function and then transfer them to the target node in the information aggregation phase. Corso et al.^33^ incorporated edge feature aggregation into the Message Passing Neural Network (MPNN) framework. The specific message passing framework is outlined below:

2 $${X}_{i}^{\left(t+1\right)}=U\left({X}_{i}^{\left(t\right)}, \begin{array}{c}\oplus \\ \left(j,i\right)\in E\end{array} M\left({X}_{i}^{\left(t\right)},{E}_{j\to i},{X}_{j}^{\left(t\right)}\right)\right)$$

The ${E}_{j\to i}$ in Eq. (2) denotes the multi-dimensional feature of edge $(j\to i)$, ${X}_{i}^{\left(t\right)}$ represents the feature representation of node $i$ in the *t*th graph convolution layer, $M$ and $U$ represent message function and vertex update function respectively, and $\oplus$ represents an aggregator that aggregates neighbor node information in some way. Equation (3) is the messaging framework of MPNN. By comparing the above two equations, we can see that Corso et al. made full use of the multi-dimensional edge features on the basis of the MPNN framework, and introduced the edge features into the information aggregation process.

3 $${X}_{i}^{\left(t+1\right)}=U\left({X}_{i}^{\left(t\right)}, \begin{array}{c}\oplus \\ \left(j,i\right)\in E\end{array} M\left({X}_{i}^{\left(t\right)},{X}_{j}^{\left(t\right)}\right)\right)$$

In order to make full use of edge information, Mahbub et al.^34^ not only introduced multi-dimensional edge features into the process of information aggregation, but also used edge features to calculate the attention coefficient between nodes, and proposed Edge Aggregated Graph Attention Networks (EGRET). Ordinary GAT^5^ only uses the features of two nodes to calculate the attention coefficient between two nodes, while EGRET not only uses the features of two nodes, but also combines the edge features between two nodes. The specific attention coefficient is calculated as follows:

4 $${e}_{ji}=\Omega \left({W}^{\alpha }\left[{W}^{\nu }{h}_{i}\Vert {W}^{\nu }{h}_{j}\Vert {W}^{\rho }{\xi }_{ji}\right]\right)$$

In the above Eq. (4), ${e}_{ji}$ represents the attention coefficient between node $i$ and $j$, ${W}^{\nu }$ and ${W}^{\rho }$ are learnable parameter matrices, ${h}_{i}$ and ${h}_{j}$ represent the feature vectors of node $i$ and node $j$, respectively. ${\xi }_{ji}$ represents the edge features of the directed edge from node $j$ to node $i$. The symbol “||” indicates the concatenation operation. $\Omega (\cdot )$ represents the activation function. In addition, EGRET also applies edge features to the process of information aggregation, and they aggregate edge features with neighbor node features. For the feature representation ${h}_{i}$ of node $i$, the final representation ${\widehat{\mathrm{h}}}_{i}$ processed by the edge aggregation graph attention layer is:

5 $${\widehat{h}}_{i}=\sigma \left(\sum_{j\in {N}_{i}}{\alpha }_{ji}{W}^{\nu }{h}_{j}+\sum_{j\in {N}_{i}}{\alpha }_{ji}{W}^{\varepsilon }{\xi }_{ji}\right)\parallel {h}_{i}$$

In the above Eq. (5), ${N}_{i}$ represents the neighbor node of node $i$.${\alpha }_{ji}$ represents a softmax normalization on $\left\{{e}_{ji}|j\in {N}_{i}\right\}$ following Bahdanau et al.^35^. ${W}^{\nu }$ and ${W}^{\varepsilon }$ represent the learnable parameter matrix, and $\upsigma (\cdot )$ denotes the activation function. The meaning of other symbols is the same as the Eq. (4). Through the above Eq. (5), we can see that EGRET applies both edge features and neighbor node features to the feature update of the central node, and makes full use of the edge information.

#### Edge embedding learning

The above methods only use the initial edge features, and cannot learn the edge features iteratively. In real-world applications, edge information may be composed of complex feature vectors, and multiple factors can influence edge features. Simple handcrafted edge features may not be sufficient to accurately capture and utilize the inter-node relationships within the graph. Consequently, these models may fail to fully exploit the information available in the graph data. To address this limitation, the following method takes multi-dimensional edge features as input and iteratively updates each layer of the graph neural network model to learn the edge embedding representation.

Inspired by the “LineGraph” in graph theory, Jiang et al.^36^ proposed Convolution with Edge-Node Switching graph neural network (CensNet). This is a kind of network that can alternately learn node embedding and edge embedding. CensNet builds an auxiliary graph by changing the nodes in the original undirected graph into edges of line graph (edges are also transformed into nodes). CensNet alternately trains the model on the original undirected graph and auxiliary graph to update node embedding and edge embedding. CensNet is different from the above methods that only learn node embeddings, it can embed both nodes and edges into the latent feature space.

Yang et al.^37^ proposed a model called NENN that incorporates node and edge features into GNN to take advantage of rich edge information. NENN adopts a hierarchical dual-level attention mechanism, and node-level attention layers and edge-level attention layers are alternately stacked to learn node embeddings and edge embeddings. Unlike CensNet, which cannot handle directed and large graphs due to approximated spectral graph convolution, NENN uses spatial domain-based graph convolution to address this limitation. NENN extends the range of adjacent nodes to the neighbors of edges, and the range of adjacent edges to the neighbors of nodes. Additionally, NENN introduces an attention mechanism to learn more effective embedding representations.

Wang et al.^38^ highlighted that the majority of GCNs are designed using single-dimensional edge features, which do not fully exploit the abundant edge information present in the graph. To address this, they proposed Multi-dimensional Edge-enhanced Graph Convolutional Networks (ME-GCN) for semi-supervised text classification. In ME-GCN, edge features are treated as multi-stream signals, where each stream performs a distinct graph convolution operation, effectively integrating rich graph edge information across the entire text corpus. In the context of skeleton-based motion recognition applications, several GCN-based models have been developed to capture adaptive correlation by constructing multiple “edge matrices”^39–41^.

## The proposed method: CEN-DGCNN

### Architecture of CEN-DGCNN

In this section, we propose a Co-embedding of Edges and Nodes with Deep Graph Convolutional Neural Network (CEN-DGCNN). We first define the notation used in this chapter: Let $G$ be a graph with $N$ nodes, the node features are represented by the $N\times F$ matrix $X$, and the edge features are represented by the $N\times N\times P$ tensor $E$. We use the index in the subscript to represent the elements of the matrix or tensor, for example, ${X}_{ij}\in R(i=1, 2, \dots , N; j=1, 2, \dots , F)$ represents the ${j}^{th}$ channel of the $F$-dimensional feature vector of the ${i}^{th}$ node in the graph $G$, and ${E}_{ijp}\in R(i,j=1, 2, \ldots , N; p=1, 2, \ldots , P)$ represents the *P*th channel of the $P$-dimensional feature vector of the edge $(i, j)$. In the subscript, we use to indicate the selection of the entire range (slice) of the corresponding dimension, for example, ${X}_{i\cdot }\in {R}^{F}$ indicates the $F$-dimensional feature vector of the ${i}^{th}$ node in graph $G$, and ${E}_{ij\cdot }\in {R}^{P}$ indicates the $P$-dimensional feature vector of the edge $(i, j)$. If the edge $(i, j)$ does not exist, we set ${E}_{ij\cdot }=[0, 0, 0, ..., 0]$. Table 1 summarizes the symbols used in this paper.

**Table 1:** Table of symbols used in this paper.

Symbol	Definition
$G=(V,E)$	$G$: input graph, $V$: node set, $E$: edge set
$\left|V\right|=N$	$N$: number of nodes
$v\in V$	Nodes in $G$
$e\in E$	Edges in $E$
$X\in {R}^{N\times F}$	Node feature matrix
$E\in {R}^{N\times N\times P}$	Multi-dimensional edge feature matrix
${x}_{i}^{(l)}\in {R}^{F}$	The F-dimensional node embedding of node $i$ at layer $l$
${e}_{ij}^{(l)}\in {R}^{P}$	The P-dimensional edge embedding of edge $ij$ at layer $l$
$N(v)$	Set of one-hop neighbors of node $v$ in $G$
$M(\cdot )$	Message aggregation function
$\sigma (\cdot )$	Activation function
$\alpha (\cdot )$	Filter constructor
$g(\cdot )$	Feature transformation function
$||$	Concatenation operation
${W}^{(l)}$	A layer-specific trainable weight matrix
${\delta }_{l}$	The weight matrix decay parameter of the ${l}^{th}$ layer
${I}_{n}$	Identity matrix
$\zeta ,\eta ,\theta$	Hyperparameters for adjusting the proportion of information aggregation
$f(i,j)$	A function used to calculate the attention coefficient between $i$ and $j$
$a$	The weight vector that projects the concatenate vector to the scalar

**Figure 2:**
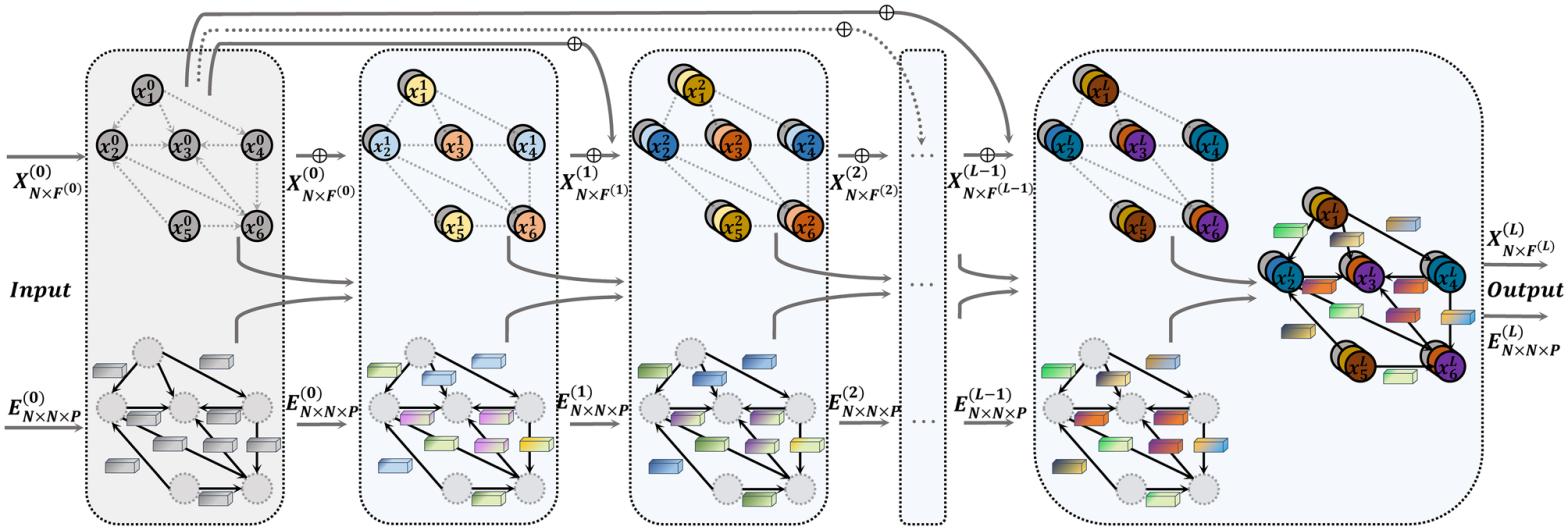
The overall architecture of Co-embedding of Edges and Nodes with Deep Graph Convolutional Neural Network (CEN-DGCNN).

Figure 2 depicts the architecture of the CEN-DGCNN model. The input graph $G$ has initial node features ${X}_{N\times {F}^{(0)}}^{(0)}$ and edge features ${E}_{N\times N\times P}^{(0)}$, where the superscript $(l)$ denotes the *l*th layer output, $N\times {F}^{(l)}$ represents the shape of the node feature matrix output by the ${l}^{th}$ layer, and $N\times N\times P$ represents the shape of the $P$-channel edge feature matrix. To reduce the influence of edge noise in the input graph, ${E}_{N\times N\times P}^{(0)}$ is pre-processed by double random normalization before it is input into CEN-DGCNN. The first CEN-DGCNN graph convolution layer generates new edge features ${E}_{N\times N\times P}^{(1)}$ from the input node features and edge features, and ${E}_{N\times N\times P}^{(1)}$ is then used as a multi-channel filter to perform graph convolution operation on ${X}_{N\times {F}^{(0)}}^{(0)}$, yielding ${X}_{N\times {F}^{(1)}}^{(1)}$. The node features and edge features output by the first graph convolutional layer are used as input for the second graph convolutional layer, where the edge features are updated to generate ${E}_{N\times N\times P}^{(2)}$, and ${E}_{N\times N\times P}^{(2)}$ is used as a multi-channel filter to perform graph convolution operations on ${X}_{N\times {F}^{(1)}}^{(1)}$ to generate ${X}_{N\times {F}^{(2)}}^{(2)}$. This process is repeated for each subsequent layer. In each graph convolutional layer, a nonlinear activation is applied to the node feature matrix, resulting in a corresponding ${F}^{(l)}$-dimensional node embedding. To extract more refined high-order features and non-local structural features of nodes, we use a deep graph convolution neural network structure, with the model depth set to 64 layers after experimental verification. To avoid over-smoothing or over-squeezing caused by model deepening, nodes in each layer aggregate a part of the initial node information and the previous layer node information in addition to the information of neighboring nodes. In Fig. 2, the concatenation operation, represented by the symbol “$\oplus$”, is used to combine the node information of the previous layer's output and the initial input. After passing through $L$ layers of CEN-DGCNN, we obtain the output graph ${G}{\prime}$ (composed of ${X}_{N\times {F}^{(L)}}^{(L)}$ and ${E}_{N\times N\times P}^{(L)}$) shown in the rightmost box in Fig. 2. The output node features can be used for downstream tasks such as node classification and graph prediction, and the output edge features can be used for tasks such as edge classification and edge prediction.

The CEN-DGCNN has several structural differences from traditional GCN. Specifically: (1) CEN-DGCNN fully utilizes edge feature information by associating edge attributes with edge feature vectors, and representing edges using multi-dimensional feature vectors, rather than the one-dimensional edge features or binary edge indicators used in ordinary GCN. (2) CEN-DGCNN employs multi-dimensional edge feature matrices (as shown in Fig. 1b) as multi-channel filters, instead of using binary adjacency matrices or one-dimensional positive matrices as single-channel filters like ordinary GCN. (3) CEN-DGCNN learns edge features as a learnable parameter that can be adjusted across layers, rather than using the same adjacency matrix at each layer as normal GCN. (4) CEN-DGCNN distinguishes itself from the shallow GCN structure by employing a deep architecture while effectively mitigating over-smoothing and over-squeezing issues. (5) CEN-DGCNN performs node embedding and edge embedding in parallel in each layer, effectively fusing node and edge features for graph convolution.

### Message passing framework

Currently, there are three primary general frameworks for graph neural networks: Message Passing Neural Network (MPNN), Non-Local Neural Network (NLNN), and Graph Network (GN). In the MPNN framework, node representations are obtained by iteratively propagating messages through the message and update functions for K rounds. While the message function proposed by MPNN^1^ aims to aggregate node information, neighboring nodes, and edges, but most GNN models based on the MPNN framework do not aggregate edge information due to the lack of edge features. The NLNN framework is a general summary of graph neural network models based on attention mechanisms, and the Graph Attention Network (GAT) can be considered a special case. The GN proposes a more comprehensive model. Because our proposed CEN-DGCNN model needs to meet the application scenarios of large-scale graphs, and needs to capture the non-local structural features of nodes, but also need to aggregate multi-dimensional edge features. Therefore, the message passing framework adopted by CEN-DGCNN needs to meet the following three requirements: (1) The ability to extract non-local structural features; (2) The ability to prevent over-smoothing; (3) The ability to aggregate multi-dimensional edge features. To meet these requirements, we propose a new graph neural network message passing framework as follows:

6 $${x}_{v}^{\left(l+1\right)}=\sigma \left(\sum_{w\in N\left(v\right)}{M}_{l}\left({x}_{v}^{\left(0\right)}, {x}_{v}^{\left(l-1\right)}, {x}_{v}^{\left(l\right)}, {x}_{w}^{\left(l\right)}, {e}_{vw}^{\left(l\right)}\right)\right)$$

**Figure 3:**
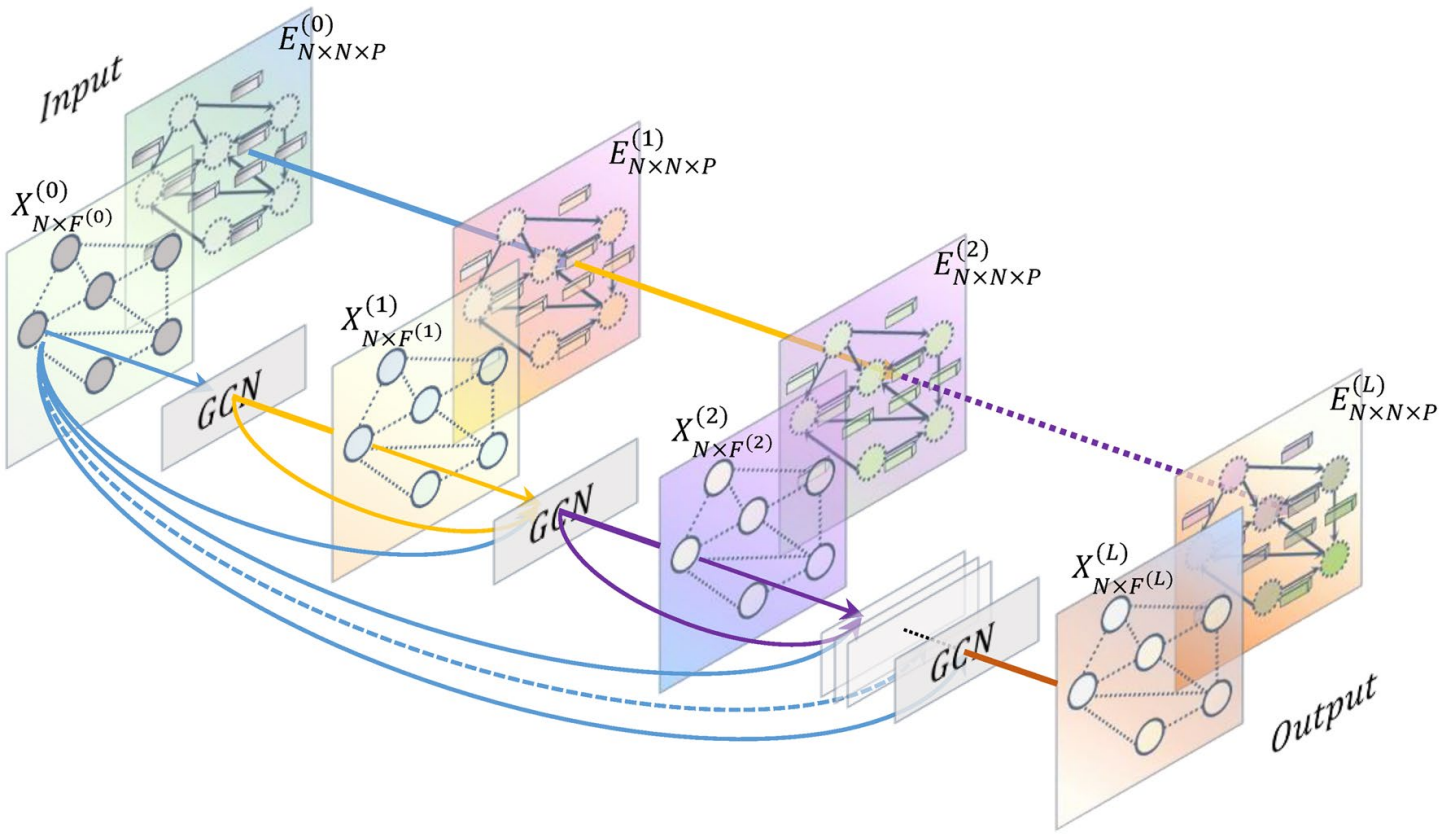
The novel graph neural network message passing framework proposed by us.

The $\sigma (\cdot )$ in the above Eq. (6) represents the activation function, and ${M}_{l}$ represents the aggregation function of the *l*th layer. The above framework is applicable to the scenario of building a deep graph convolutional network model, and can simultaneously aggregate neighbor node features and edge features to the central node. It can be seen from the above equation that the (*l* + 1)th layer of node feature ${x}_{v}^{(l+1)}$ aggregates the initial node feature ${x}_{v}^{\left(0\right)}$, the node feature ${x}_{v}^{\left(l-1\right)}$ of (*l* + 1)th layer, the node feature ${x}_{v}^{\left(l\right)}$ of *l*th layer, as well as the neighbor node features ${x}_{w}^{\left(l\right)}$ of *l*th layer and all edge features ${e}_{vw}^{(l)}$ connected to node $v$. We adopt the idea of residual and dense connections to aggregate the initial and previous layer's features, effectively alleviating over-smoothing and increasing network depth by connecting outputs across layers. Our proposed novel message passing framework is shown in Fig. 3. We iteratively apply graph convolution to aggregate the features of remote nodes and obtain non-local structural and high-order node features. Additionally, we aggregate multi-dimensional edge features during the graph convolution process, which will be elaborated on in the following section.

The new framework can simultaneously aggregate neighbor node information and multi-dimensional edge features to the central node, and is suitable for the construction of deep graph convolutional neural network models. This framework draws lessons from the idea of residual connection and dense connection, and each layer node feature aggregates part of the initial node feature and the previous layer node feature, which effectively avoids the problem of over-smoothing. The framework has the following three characteristics: (1) The node non-local structural features and more refined high-order features can be obtained; (2) Over-smoothing problem can be effectively avoided; (3) Multi-dimensional edge features can be aggregated to the central node.

### Graph convolution layer

In this section, we outline the graph convolution layers of CEN-DGCNN. Traditional GCN models only regard edges as binary indicator variables or one-dimensional real values, completely ignoring the rich information contained in edges. In a large number of application scenarios, edges contain information such as attributes, types, and connection strengths. The traditional graph neural network cannot express rich edge information, nor can it incorporate edge information into the model. And each layer of the traditional GCN model uses the original adjacency matrix as a single-channel filter for node feature filtering. This will bring two problems: First, the original adjacency matrix may contain noise and is not optimal for filtering; Second, the edge features are not fully incorporated into the model. Although complex graph convolutional models can extract fine node features, repeated use of a simple adjacency matrix that may contain noise to filter each layer of node features will limit the effectiveness of the filtering operation. To address these issues, we introduce the following information aggregation operations based on the new messaging framework proposed in the previous section:

7 $${X}^{\left(l\right)}=\sigma \left[\begin{array}{c}P\\ \parallel \\ p=1\end{array}\left({M}_{l}\left({\alpha }_{\cdot \cdot p}^{\left(l\right)}\left({X}^{\left(l-1\right)}, {E}_{\cdot \cdot p}^{\left(l-1\right)}\right){g}^{\left(l\right)}\left({X}^{\left(l-1\right)}\right), {g}^{\left(l\right)}\left({X}^{\left(l-2\right)}\right), {g}^{\left(l\right)}\left({X}^{\left(0\right)}\right)\right)\right)\right]$$

The above Eq. (7) defines the output of the *l*th layer graph convolution, i.e., the node feature matrix output by the *l*th layer. Where $\sigma$ represents the nonlinear activation function, ${M}_{l}$ represents the aggregation function of the *l*th layer, $\alpha$ is the function used to generate a multi-channel filter with shape of $N\times N\times P$, and ${\alpha }_{\cdot \cdot p}$ represents the slice of the *p*th channel of the multi-channel filter. Moreover, ${g}^{\left(l\right)}$ represents the node feature transformation function of the *l*th layer, while the concatenation of the node feature slices of $P$ channels is indicated by the symbol “$||$”.

For the feature transformation function $g$, a linear mapping as shown in Eq. (8) is usually used, but the frequent interaction between different dimensions of the feature matrix degrades the performance of the model^42^. Therefore, adopting linear maps as feature transformation functions is not suitable for our proposed deep graph convolutional model. In order to ensure that the frequent interaction between different dimensions of the feature matrix in the deep model will not degrade the model performance, we adopt the identity mapping mechanism^25^ as shown in Eq. (9) for the feature transformation function $g$. The idea of identity mapping is to add the identity matrix to the weight matrix $W$ in a certain proportion, and the weight of the identity matrix will increase as the model deepens. ${\delta }_{l}$ in Eq. (9) is the weight matrix attenuation that changes with the number of layers parameter.

8 $${g}^{\left(l\right)}\left(X\right)={W}^{\left(l\right)}X$$

9 $${g}^{\left(l\right)}\left(X\right)=\left(\left(1-{\delta }_{l}\right){I}_{n}+{\delta }_{l}{W}^{\left(l\right)}\right)X$$

10 $${\delta }_{l}=\mathrm{log}\left(\frac{0.5}{l}+\lambda \right)$$

For the aggregation function ${M}_{l}$, our definition is as Eq. (11), where $\upzeta$, $\eta$, and $\theta$ represent the weight parameters of the corresponding variables respectively. By adjusting the weight parameters, the three variables can be aggregated according to different weights.

11 $${M}_{l}\left({X}_{1}, {X}_{2}, {X}_{3}\right)=\upzeta {X}_{1}+\eta {X}_{2}+\theta {X}_{3}$$

Our proposed graph convolution layer can effectively learn multi-dimensional edge features and incorporate them into the process of information aggregation, allowing for full utilization of edge information. The model utilizes a new multi-channel filter, enabling graph convolution operations on different channels of edge features. Additionally, the filter can also reduce noise, with specific methods to be discussed in the next section.

### Edge feature update based on attention mechanism

**Figure 4:**
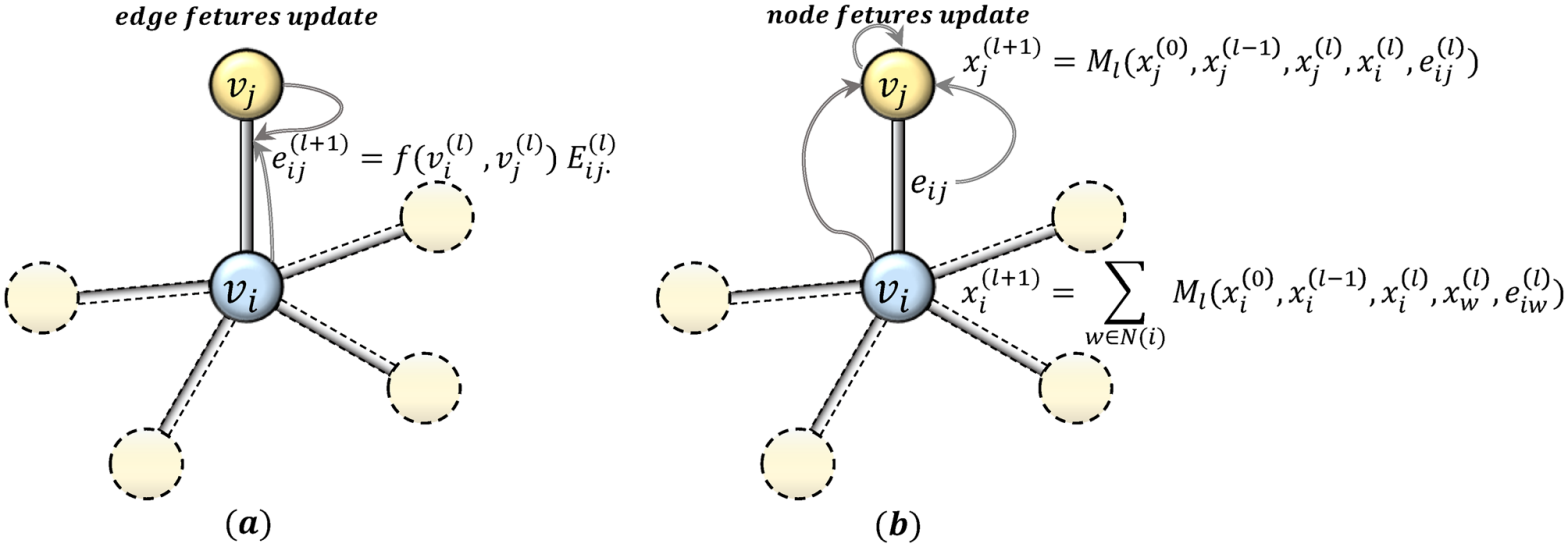
Edge features update and node features update of CEN-DGCNN.

This section will describe the learning of multi-dimensional edge features and the construction of multi-channel filters. As shown in Fig. 4a, the edge feature ${e}_{ij}$ of the (*l* + 1)th ${(l+1)}^{th}$ layer is updated according to the features of the two nodes it connects. At the same time, as shown in Fig. 4b, the node will aggregate its neighbor node features and edge features to update its own features. The above edge feature update and node feature update are performed simultaneously in the same graph convolutional layer.

The ${\alpha }^{\left(l\right)}$ function in Eq. (7) is used to generate the multi-channel filter of the *l*th layer of the model, and the $\alpha$ function uses the attention mechanism to construct the filter. The attention mechanism adopted in most existing GNN models is improved based on GAT, and the attention coefficient in GAT only depends on the features of nodes at both ends of the edge. In order to make full use of edge information, the fusion of node features and edge features is realized. CEN-DGCNN uses the edge features and the two node features connected by the edge to learn the attention coefficient. Since the multi-dimensional edge features we adopt have multiple feature channels, we conduct separate attention learning for each channel. Assuming that we construct a single-channel filter for the *p*th dimension of the edge feature with $P$-dimensional feature channels, then the filter ${\alpha }_{\cdot\cdot p}^{\left(l\right)}$ of the *p*th feature channel of the model is a function of the previous layer node feature ${X}^{\left(l-1\right)}$ and the previous layer edge feature ${E}_{\cdot\cdot p}^{\left(l-1\right)}$. We define the filter based on attention mechanism as follows:

12 $${\alpha }_{ijp}^{\left(l\right)}\left({X}^{\left(l-1\right)}, {E}_{ijp}^{\left(l-1\right)}\right)=\frac{\mathrm{exp}\left({L}_{ReLU}({a}^{T}[W{X}_{i\cdot }^{\left(l-1\right)}||W{X}_{j\cdot }^{\left(l-1\right)}])\right)}{{\sum }_{k\in {N}_{i}}\mathrm{exp}\left({L}_{ReLU}({a}^{T}[W{X}_{i\cdot }^{\left(l-1\right)}||W{X}_{k\cdot }^{\left(l-1\right)}])\right)}{E}_{ijp}^{\left(l-1\right)}$$

The above equation defines the filtering parameters for the *p*th channel filter of the *l*th graph convolutional layer in CEN-DGCNN. Where ${X}_{i\cdot }$ and ${X}_{j\cdot }$ represent the feature vectors of node $i$ and node $j$, respectively. $W$ is a learnable parameter matrix that adjusts the output dimension of node feature vectors. The symbol “$||$” represents the concatenation operation, which is used to concatenate two vectors. The weight vector ${a}^{T}$ projects the concatenated vector to a scalar. ${L}_{ReLU}$ represents the $LeakyReLU$ activation function. We also apply regularization to the function used to compute the attention coefficient. We calculate the attention coefficient for each edge feature channel and then multiply it by the corresponding edge feature matrix. Each graph convolution layer updates the attention coefficient and edge features according to the new node features. The update equation for the edge feature matrix is given as follows:

13 $${\widehat{E}}^{\left(l\right)}={\alpha }^{\left(l\right)}$$

Since the edge feature will generate random noise during the learning process, and the graph data itself in various application scenarios may also have noise, so we need to denoise the edge feature matrix learned by each layer. Wang et al.^43^ proposed to use doubly stochastic matrices for network enhancement and confirmed the network denoising performance of doubly stochastic matrices. Experiments prove that the network enhancement method they proposed removes weak edges, enhances real connections, and makes downstream tasks perform better. To improve the performance of CEN-DGCNN, we apply doubly stochastic normalization to the edge feature matrix of each layer, and the final edge feature matrix E obtained after normalization is as follows:

14 $${\widetilde{E}}_{ijp}=\frac{{\widehat{E}}_{ijp}}{{\sum }_{k=1}^{N}{\widehat{E}}_{ikp}}$$

15 $${E}_{ijp}=\sum_{k=1}^{N}\frac{{\widetilde{E}}_{ikp}{\widetilde{E}}_{jkp}}{{\sum }_{v=1}^{N}{\widetilde{E}}_{vkp}}$$

After the edge feature matrix undergoes a doubly stochastic normalization operation, the sum of the rows and columns of each feature channel in the edge feature matrix ${E}_{\cdot \cdot p}$($p=1, 2, \dots ,\mathrm{ P}$) is 1, indicating that the matrix is left-stochastic and right-stochastic. The CEN-DGCNN we proposed has a deep structure, and the edge feature matrix will be multiplied multiple times across layers, and the normalized doubly stochastic matrix will make the cross-layer update process more stable.

## Discussion

As previously mentioned, our objective is to develop a graph neural network model that can effectively learn multi-dimensional edge features while simultaneously learning node features and edge features in the same graph convolution layer. To extract finer non-local structural features of nodes and classify nodes into different categories as accurately as possible, we aim to construct a deep graph convolutional network model. However, when the network is too deep, over-smoothing or over-squeezing will occur, causing the features of all nodes to tend to be consistent, and the nodes will become indistinguishable. Additionally, we require the GNN model to be capable of handling directed graphs to meet the requirements of application scenarios that involve directed graphs.

To address the above issues, we propose CEN-DGCNN, a deep feed-forward graph convolutional network model that utilizes multi-dimensional edge feature vectors instead of the traditional adjacency matrix as a node information filter. We use multi-dimensional edge feature vectors to construct multi-channel filters to better capture node features. In each graph convolutional layer of CEN-DGCNN, node embedding learning and edge embedding learning are carried out simultaneously, and respective model architectures are used to learn node features and edge features. Furthermore, the node features and edge features learned in parallel in each layer are used mutually to assist in learning edge features and node features respectively. For node information aggregation and update, we employ a deep GCN structure to extract non-local structural features and high-order features of nodes by mining long-range dependencies between them. To avoid the over-smoothing and over-squeezing problems associated with GCN, we introduce the concepts of residual and dense connections in the node information aggregation process, and adopt identity mapping for linear transformations. Multi-dimensional edge features can be flexibly utilized to design graph convolution filters, and we propose multi-channel filters that efficiently handle directed graph data. With these techniques, CEN-DGCNN achieves impressive results.

Our research has certain limitations. The inclusion of multi-dimensional edge features increases the number of parameters, making it challenging for CEN-DGCNN to handle large-scale networks. In our future work, we will focus on enhancing the model and investigating strategies to apply CEN-DGCNN effectively in large-scale network applications.

## Experiments

In this section, we will conduct node classification and link prediction tasks on various datasets, and compare the results with multiple baseline methods to demonstrate that CEN-DGCNN effectively captures more precise node features through multi-channel filters constructed by multi-dimensional edge feature matrices. Moreover, we will conduct ablation experiments to validate the significance and necessity of each component in CEN-DGCNN. Furthermore, we will quantitatively assess the smoothness of each layer in the model to establish that CEN-DGCNN effectively mitigates over-smoothing while adopting a deep architecture. Lastly, we will construct a directional multi-channel filter tailored to the dataset's characteristics to demonstrate the superiority of the multi-channel filter based on multi-dimensional edge features in node classification. The effectiveness of multi-dimensional edge features will be confirmed by comparing various edge feature encoding methods.

### Model settings

All experiments run on a computer with 12-core Intel(R) i7-12700KF CPU, 16 GB RAM, and NVIDIA GeForce RTX 3080 12 GB GPU. We use PyTorch to implement our methods. We implemented the construction of a 64-layer CEN-DGCNN model, with the output dimensions of the input layer and all intermediate hidden layers set to 64 dimensions. We use the ADAM optimizer^44^ with a learning rate of 0.005 and a weight decay parameter of 0.0005 for model optimization. Dropout^30^ with a rate of 0.2 is applied to the input and output features of the model during training. The batch size is set to 20, and the maximum number of epochs is set to 10,000, with early stopping after 150 epochs of non-decreasing validation loss. We use the LeakyReLU activation function^45^ with a slope of 0.2 for the hidden layers. The parameter λ in the weight matrix decay parameter of Eq. (10) is set to 0.5, while ζ, η, and θ in Eq. (11) are learnable weight coefficients, and the model will learn automatically during the backpropagation process. The data set partitioning for the three citation networks follows the standard split^2,27,42^. As for the disease spreading network and flight network, we adopt the division method described in related work^46^. We report the average accuracy over 20 runs.

### Node classification for directed graphs

In this section, we will encode multi-dimensional edge features for directed graphs and construct multi-channel filters by learning direction-related multi-dimensional edge features. The aim is to obtain more accurate node features. Subsequently, we will compare the node classification results with various baseline methods.

#### Dataset

In this section, we assess the performance of CEN-DGCNN on the node classification task across five datasets, which comprise three citation networks, a disease spreading network, and a flight network. Table 2 provides a comprehensive overview of the specific parameters associated with each dataset. A brief introduction is provided for each dataset below.

1. Cora dataset is a citation network composed of machine learning related papers, and it is a commonly used dataset for node classification tasks. The dataset consists of 2708 nodes, each node represents a paper, and all papers are divided into seven categories: Case Based, Genetic Algorithms, Neural Networks, Rule Learning, Reinforcement Learning, Probabilistic Methods, Theory. The features of each paper are represented by a 1433-dimensional word vector, and each dimension represents a keyword in the field of machine learning. Each paper in Cora cites at least one other paper, or is cited by another paper. Cora is a digraph with a total of 5429 reference relationships.
2. The Citeseer citation network dataset contains 3327 papers, and the features of each paper are represented by 3703-dimensional word vectors. There are six categories of papers, namely: Agents, IR, ML, DB, AI, HCI. Citeseer has a total of 4732 citation relations, which are also digraphs.
3. The PubMed citation dataset consists of 19717 scientific publications on diabetes from the Pubmed database, divided into three categories, where each publication is described by a TF/IDF weighted word vector in a dictionary of 500 unique words. In this dataset, a total of 44338 edges are present, representing directed citation relationships between publications.
4. The Disease dataset simulates the SIR disease transmission model. It consists of 1044 nodes representing individuals classified into either infected or uninfected states. Each node is characterized by a 1000-dimensional feature representing individual susceptibility. Additionally, the dataset includes 1043 edges that represent the propagation paths of the disease between individuals.
5. The Airport dataset simulates airport routes, where nodes represent airports. The characteristics of each airport are described by four dimensions: latitude, longitude, height information, and the GDP of the country/region to which the airport belongs. The population of the country where the airport is located serves as the classification label of the node (airport), and airports are divided into four categories accordingly. The dataset includes 18631 edges, which represent the directed routes connecting different airports.

**Table 2:** Dataset statistics.

Dataset	Graph	Nodes	Node Features	Edges	Classes
Cora	1	2708	1433	5429	7
Citeseer	1	3327	3703	4732	6
Pubmed	1	19717	500	44338	3
Disease	1	1044	1000	1043	2
Airport	1	3188	4	18631	4

#### Multi-dimensional edge feature encoding

The above data sets contain rich node features, but all data sets are directed graphs, the edges only contain direction information, and the direction of edges also contains important information about graph data. Many previous studies have treated the above benchmark data sets as undirected graphs. Therefore, in order to verify the effectiveness of our proposed CEN-DGCNN, we will encode the multi-dimensional edge features according to the direction of the edge. We encode the directed multi-dimensional edge feature vector ${E}_{ij\cdot }$ as:

16 $${E}_{ij\cdot }=\left[{E}_{ij}+{E}_{ji}, {E}_{ij}, {E}_{ji}\right]$$

In accordance with Eq. (16), we encode the edge features into three distinct edge feature channels. For the three citation datasets, namely Cora, Citeseer, and Pubmed, the three edge feature channels respectively represent the citation relationship, citations of other papers, and being cited by others. As for the Disease dataset, the three edge feature channels denote the transmission relationship, transmission to others, and transmission by others, respectively. Likewise, for the Airport dataset, the three edge feature channels signify the existence of a route, flight routes, and return routes, respectively.

The three-channel filter, constructed based on this edge feature coding method, effectively aggregates three types of neighboring node information. Comparative analysis of the baseline and ablation experiments reveals that this multi-dimensional edge feature coding method significantly enhances the model's performance compared to commonly used undirected graph processing approaches.

#### Baseline

We perform an extensive comparison of CEN-DGCNN against three categories of state-of-the-art baseline methods, namely GNN-based methods, deep architecture-based methods, and approaches involving edge embedding learning. The GNN-based methods under consideration encompass GCN^2^, GAT^5^, AMC-GCN^47^, NIGCN^48^. As for the deep architecture-based approaches, we evaluate DropEdge^31^; NodeNorm^8^, GCNII^25^, GDC^49^, DeepGWC^50^. Additionally, we include methods employing edge embedding learning, which comprise CensNet^36^; NENN^37^; EGAT^50^.

#### Result

We present a comprehensive performance comparison of CEN-DGCNN against other baseline methods in Table 3. To assess the node classification task, we evaluate the models based on the F1 score. Given that our proposed CEN-DGCNN adopts a deep graph convolutional network architecture and incorporates edge embedding learning methods, our baseline methods consist of three categories: GNN model, deep GNN model, and GNN model capable of learning edge embeddings. For a fair comparison, we maintain the same CEN-DGCNN network structure across all datasets. It is important to emphasize that we do not introduce additional edge information into the model; rather, we utilize the directed edges present in the benchmark datasets to encode multi-dimensional edge features. Consequently, the comparison of our proposed CEN-DGCNN with other baseline methods is conducted in a fair manner. The experimental results presented in Table 3 demonstrate that CEN-DGCNN, with its singular structure, attains the best results across all five datasets.

**Table 3:** Comparison of node classification accuracy with other GNN methods (highest accuracy highlighted in bold).

Method	Cora	Citeseer	Pubmed	Disease	Airport
GCN^2^	81.5 ± 0.5	70.4 ± 0.4	78.1 ± 0.4	69.8 ± 0.5	81.4 ± 0.6
GAT^5^	83.0 ± 0.5	71.6 ± 0.8	78.2 ± 0.4	70.4 ± 0.5	81.6 ± 0.4
AMC-GCN^47^	84.8 ± 0.4	72.8 ± 0.5	78.9 ± 0.3	70.8 ± 0.2	80.5 ± 0.5
NIGCN^48^	82.1 ± 1.1	71.4 ± 0.8	80.9 ± 2.0	68.5 ± 1.5	82.1 ± 1.1
DropEdge^31^ (64 layers)	78.9 ± 0.3	65.1 ± 0.5	76.9 ± 0.6	69.7 ± 1.6	82.8 ± 1.5
NodeNorm^8^ (64 layers)	83.4 ± 0.6	73.8 ± 0.8	80.4 ± 1.2	69.6 ± 0.8	83.9 ± 1.2
GCNII^25^ (64 layers)	85.5 ± 0.4	73.4 ± 0.2	79.7 ± 0.3	71.3 ± 0.4	84.5 ± 0.5
GDC^49^	83.8 ± 0.2	73.3 ± 0.3	79.9 ± 0.3	70.2 ± 0.1	83.6 ± 0.2
DeepGWC^50^ (64 layers)	86.4 ± 0.2	74.9 ± 0.5	80.7 ± 0.2	70.8 ± 0.7	84.7 ± 1.3
CensNet^36^	79.4 ± 1.0	62.5 ± 1.5	69.9 ± 2.1	64.4 ± 2.1	78.6 ± 1.8
NENN^37^	82.6 ± 0.1	68.2 ± 0.1	77.7 ± 0.1	67.7 ± 0.1	79.8 ± 0.1
EGAT^51^	82.1 ± 0.7	70.3 ± 0.5	78.1 ± 0.4	69.1 ± 0.6	80.4 ± 0.5
**CEN-DGCNN (Ours)**	**87.1 ± 0.5**	**75.0 ± 0.8**	**81.8 ± 0.4**	**73.5 ± 0.6**	**85.8 ± 0.6**

#### Analysis

(1) By comparing CEN-DGCNN with the deep GNN baseline method, we observe its superior performance over all deep GNN baseline methods. CEN-DGCNN introduces the learning of multi-dimensional edge features within the context of a deep graph convolutional network architecture, utilizing the multi-dimensional edge feature matrix for constructing node feature filters. Thus, we can conclude that GNNs can derive substantial benefits from the integration of multi-dimensional edge features. (2) In comparison to the three GNN baseline methods employing learnable edge embeddings, CEN-DGCNN demonstrates enhanced performance. CEN-DGCNN incorporates the deep graph convolutional neural network architecture while simultaneously learning multi-dimensional edge embeddings. Hence, we deduce that the deep model architecture significantly contributes to the performance improvement of graph convolutional neural networks. (3) Distinguished from the prevalent neighborhood message passing method in GNN, CEN-DGCNN adopts a new message passing framework, and realizes non-local message passing through dense connections. As can be seen from the results of CEN-DGCNN outperforming all 12 baseline methods, our proposed new non-local message passing framework is very effective in learning node feature representations.

### Link prediction

In this section, we aim to further validate the performance of CEN-DGCNN through the link prediction task. We evaluate CEN-DGCNN's link prediction performance on five datasets, including three citation networks, the disease spreading network, and the flight network. As baseline methods, we select seven models known for their state-of-the-art performance, and we adopt the experimental setup of VGAE^52^. Specifically, we use 85% of the edges as the training set, 5% as the validation set, and 10% as the test set. To generate negative samples (unconnected false edges) for the validation and test sets, we randomly sample 5% and 10% of the edges, respectively. We use the area under the ROC curve (AUC) as the evaluation metric for link prediction. All other settings of CEN-DGCNN remain consistent with the previous experiments.

The experimental results are presented in Table 4, indicating that CEN-DGCNN, employing the non-local message passing framework and the edge-node co-embedding learning structure, outperforms other methods on the five datasets. Previous studies by Kipf et al.^2^ suggested that deeper graph neural networks might underperform compared to shallow networks, and some research^53^ demonstrated that using two-layer GCNs as encoders is a common approach for link prediction tasks. However, with the integration of our proposed non-local message passing framework, CEN-DGCNN achieves a 64-layer model structure, successfully overcoming the degradation issue associated with excessively deep models and surpassing the performance of shallow layers. Hence, we can assert that incorporating deep model architecture and multi-dimensional edge features contributes to the enhancement of GCNs’ performance.

**Table 4:** Comparison of link prediction accuracy with other GNN methods (highest accuracy highlighted in bold).

Method	Cora	Citeseer	Pubmed	Disease	Airport
GCN^2^	90.5 ± 0.2%	82.6 ± 0.4%	89.6 ± 3.6%	58.0 ± 1.4%	89.3 ± 0.4%
GAT^5^	93.2 ± 0.2%	86.5 ± 1.5%	91.5 ± 1.8%	58.2 ± 0.9%	90.8 ± 0.2%
SAGE^54^	85.5 ± 0.5%	82.2 ± 0.2%	86.2 ± 0.9%	65.9 ± 0.3%	90.4 ± 0.5%
DeepWalk^55^	83.1 ± 0.1%	80.5 ± 0.1%	84.4 ± 0.0%	59.8 ± 0.2%	88.4 ± 0.3%
VGAE^52^	91.4 ± 0.1%	90.8 ± 0.1%	94.4 ± 0.1%	70.5 ± 0.5%	91.4 ± 0.2%
CensNet-VAE^36^	91.7 ± 0.1%	90.6 ± 0.1%	93.5 ± 0.1%	-	-
SGC^56^	91.5 ± 0.2%	89.6 ± 0.2%	94.1 ± 0.1%	65.3 ± 0.3%	89.8 ± 0.3%
**CEN-DGCNN** (ours)	**92.63%**	**91.24%**	**94.42%**	**72.30%**	**92.1%**

### Node classification results on large-scale datasets

In this section, we assess the performance of CEN-DGCNN in the context of node classification tasks conducted on large-scale datasets. Given CEN-DGCNN's capability to encode multi-dimensional features based on edge directionality, we have selected the directed graph dataset ogbn-arxiv from the Open Graph Benchmark^57^ (OGB) for evaluation. Additionally, to underscore the efficacy of encoding edge features with respect to edge directionality, we have included the large-scale undirected graph dataset Reddit for assessment. Table 5 provides an overview of the specific parameters for these two extensive datasets.

**Table 5:** Dataset statistics.

Dataset	Graph	Nodes	Node Features	Edges	Classes	Metrics
ogbn-arxiv	1	169,343	128	1,166,243	40	ACC
Reddit	1	232,965	602	11,606,919	41	ACC

#### Model settings

In terms of model configuration, our primary objective is to conduct a comprehensive comparison with Li et al.’s RevGNN^58^, where ‘RevGAT-Deep’ signifies a deep version featuring 28 layers and 128 channels, while 'RevGAT-Wide' represents the wide version with 5 layers and 1068 channels. This comparison is undertaken to underscore the distinctive advantages of CEN-DGCNN.

Hence, for the experiments in this section, we adopt a model architecture comprising 5 layers and 64 hidden channels. The model is trained for a maximum of 200 epochs, and we implement an early stopping strategy. Specifically, training ceases if the validation set's loss fails to decrease over a consecutive span of 10 epochs. This configuration is devised to demonstrate that, even with a limited number of hidden channels, CEN-DGCNN can still achieve outstanding results through the utilization of multi-dimensional edge feature encoding, thereby showcasing its superiority. For the ogbn-arxiv dataset, we employ the identical dataset partitions as prescribed in the OGB open benchmark, which consists of 54% for training, 18% for validation, and 28% for testing. In the case of the Reddit dataset, we utilize the standard data divisions, comprising 66% for training, 10% for validation, and 24% for testing.

For the implementation of multi-dimensional edge feature encoding, we tailor our approach to the citation direction within the ogbn-arxiv dataset, with a comprehensive description provided in Section “Multi-dimensional edge feature encoding”. Within this encoding process, we employ three distinct edge feature channels to encapsulate the citation relationship, the citations from other research papers, and the instances of being cited by others. In the context of the Reddit dataset, characterized by an undirected graph, we transform Reddit’s edge features into a standard 1-dimensional adjacency matrix. In this matrix, the edge channel serves as an indicator, signifying whether two posts have received comments from the same user, thereby reflecting the degree of correlation between them. The remaining model configurations adhere to the specifications outlined in Section “Model settings”.

#### Results and analysis

Table 6 provides a detailed performance comparison of CEN-DGCNN with various baseline methods across two extensive datasets. In this table, we employ bold formatting to highlight the best-performing results. It is evident from the table that CEN-DGCNN demonstrates outstanding performance on the sizeable citation dataset, ogbn-arxiv, while also achieving commendable results on the Reddit dataset.

**Table 6:** Results on the ogbn-arxiv and Reddit datasets (highest accuracy highlighted in bold).

Models	Accuracy (%)	
	ogbn-arxiv	Reddit
GCN^2^	72.37 ± 0.10	94.46 ± 0.40
ClusterGCN^59^	71.29 ± 0.44	95.68 ± 0.03
DeeperGCN^28^	71.92 ± 0.16	–
GAT^5^	72.95 ± 0.14	–
GraphSAGE^54^	71.98 ± 0.17	96.39 ± 0.03
SIGN^60^	71.79 ± 0.08	96.12 ± 0.05
SUGAR^61^	72.22 ± 0.14	96.01 ± 0.03
AGDN^62^	73.75 ± 0.21	–
Graph Partition Soup^63^	72.35 ± 0.19	**96.41 ± 0.08**
RevGCN-Deep^58^	73.01 ± 0.31	**–**
RevGAT-Wide^58^	74.05 ± 0.11	–
**CEN-DGCNN** (ours)	**74.86 ± 0.21**	96.13 ± 0.04

To begin, we conduct a comparative analysis between RevGCN-Deep and RevGAT-Wide. RevGCN-Deep represents the deep version of the model, comprising 28 layers, each with 128 channels. Conversely, RevGAT-Wide represents the wide-body variant, characterized by a 5-layer model structure, with each layer accommodating up to 1068 channels. Through our experimental findings, it becomes evident that RevGAT-Wide exhibits superior performance compared to RevGCN-Deep. This observation suggests that increasing the number of channels contributes to enhanced performance within the RevGNN model, whereas reducing the number of layers does not lead to performance degradation. Consequently, we infer that the performance of the RevGNN model is predominantly influenced by the configuration of channel quantity.

Subsequently, we embark on a comprehensive analysis of our experimental results: (1) In contrast to RevGNN, which treats the ogbn-arxiv dataset as an undirected graph, our CEN-DGCNN encodes edges based on the citation direction within the dataset. Our experimental findings reveal that, with the same model depth, CEN-DGCNN outperforms RevGAT-Wide while utilizing only approximately 6% of the channel capacity. This serves as further evidence of the effectiveness of multi-dimensional edge feature encoding, showcasing its capacity to deliver superior results even with a reduced channel count. (2) Given that the Reddit dataset is inherently an undirected graph, we do not employ multi-dimensional edge feature encoding for this dataset. Nevertheless, the exceptional performance of multi-dimensional edge feature encoding on the ogbn-arxiv datasets, coupled with the results from the ablation experiments in Section “Different edge feature encoding methods”, provides compelling evidence of the significant impact of multi-dimensional edge feature encoding on overall model performance enhancement.

We conducted a comparative analysis between CEN-DGCNN and the top-performing baseline model, RevGAT-Wide. As discussed in detail in Section “Model complexity analysis” complexity analysis, increasing the number of channels results in an exponential increase in model parameter complexity and time complexity (it's worth noting that the complexity analysis indicates only linear growth in the number of layers). By employing a reduced channel count, we can significantly curtail the number of parameters and, theoretically, the training time. This substantial reduction in parameters and training time is achieved without compromising model performance. Therefore, in comparison to the RevGNN model, the remarkable advantage of CEN-DGCNN is that it can maintain the model accuracy while greatly reducing the number of channels, and significantly reduce the parameter complexity and time complexity.

### Quantitative and qualitative analysis of node representation smoothness

#### Quantitative analysis evaluation metric

Metric for Smoothness (MAD) is a quantitative metric proposed by Chen et al.^64^ to measure smoothness. The reason for node over-smoothing is that the model is too deep, and after many graph convolution operations, each node almost aggregates the information of the global node to itself, which leads to the consistency of the features of all nodes, i.e., the spatial distribution of node features becomes very close. Therefore, the principle of MAD is to measure node smoothness by calculating the average of the average distance of nodes to other nodes. The specific equation for calculating MAD is as follows:

17 $$MAD^{{tgt}} = \frac{{\mathop \sum\nolimits_{{i = 0}}^{n} \bar{D}_{i}^{{tgt}} }}{{\mathop \sum\nolimits_{{i = 0}}^{n} u\left( {\bar{D}_{i}^{{tgt}} } \right)}}$$

18 $$\bar{D}_{i}^{{tgt}} = \frac{{\mathop \sum \nolimits_{{j = 0}}^{n} D_{{ij}}^{{tgt}} }}{{\mathop \sum \nolimits_{{j = 0}}^{n} 1\left( {D_{{ij}}^{{tgt}} } \right)}}$$

19 $${D}^{tgt}=D \circ {M}^{tgt}$$

20 $${D}_{ij}=1-\frac{{H}_{i,:}\cdot {H}_{j,:}}{\left|{H}_{i,:}\right|\cdot \left|{H}_{j,:}\right|} i,j\in \left\{\mathrm{1,2},\dots ,n\right\}$$

The ${MAD}^{tgt}$ in the above Eq. (17) represents the MAD value of the target node pair, where $u\left(x\right)=1$ if $x>0$ otherwise $0$. ${\overline{D} }_{i}^{tgt}$ is used to calculate the average of the non-zero elements in each row of ${D}^{tgt}$. ${M}^{tgt}$ in the Eq. (19) represents an $N\times N$ mask matrix, and ○ represents an information filtering operation that uses the mask matrix ${M}^{tgt}$ to multiply the $N\times N$ distance matrix $D$ element-by-element. The element calculation of the distance matrix $D$ is shown in Eq. (20), $H$ represents the node feature matrix, and ${H}_{i,:}$ represents the feature vector of node $i$. The element values of the distance matrix $D$ are obtained by calculating the cosine value between the node pairs. It should be pointed out here that the node feature matrix $H$ is the output of the last layer of CEN-DGCNN.

#### Quantitative analysis

In order to enable CEN-DGCNN to learn multi-dimensional edge embeddings and aggregate long-range high-order node features, we propose a novel message passing framework. Based on this framework, a deep GCN model can be constructed. However, the common model is shallow structure. If the model is too deep, it will cause serious node over-smoothing problems. In order to eliminate the problem of node over-smoothing, we introduce the idea of residual connection and dense connection, and use identity mapping to transform node features. Through the above techniques, the problem of node over-smoothing caused by too deep graph neural network model is effectively solved. In this section, we quantitatively measure the node smoothness of CEN-DGCNN with a depth of 256 layers to demonstrate the effectiveness of our proposed method.

**Figure 5:**
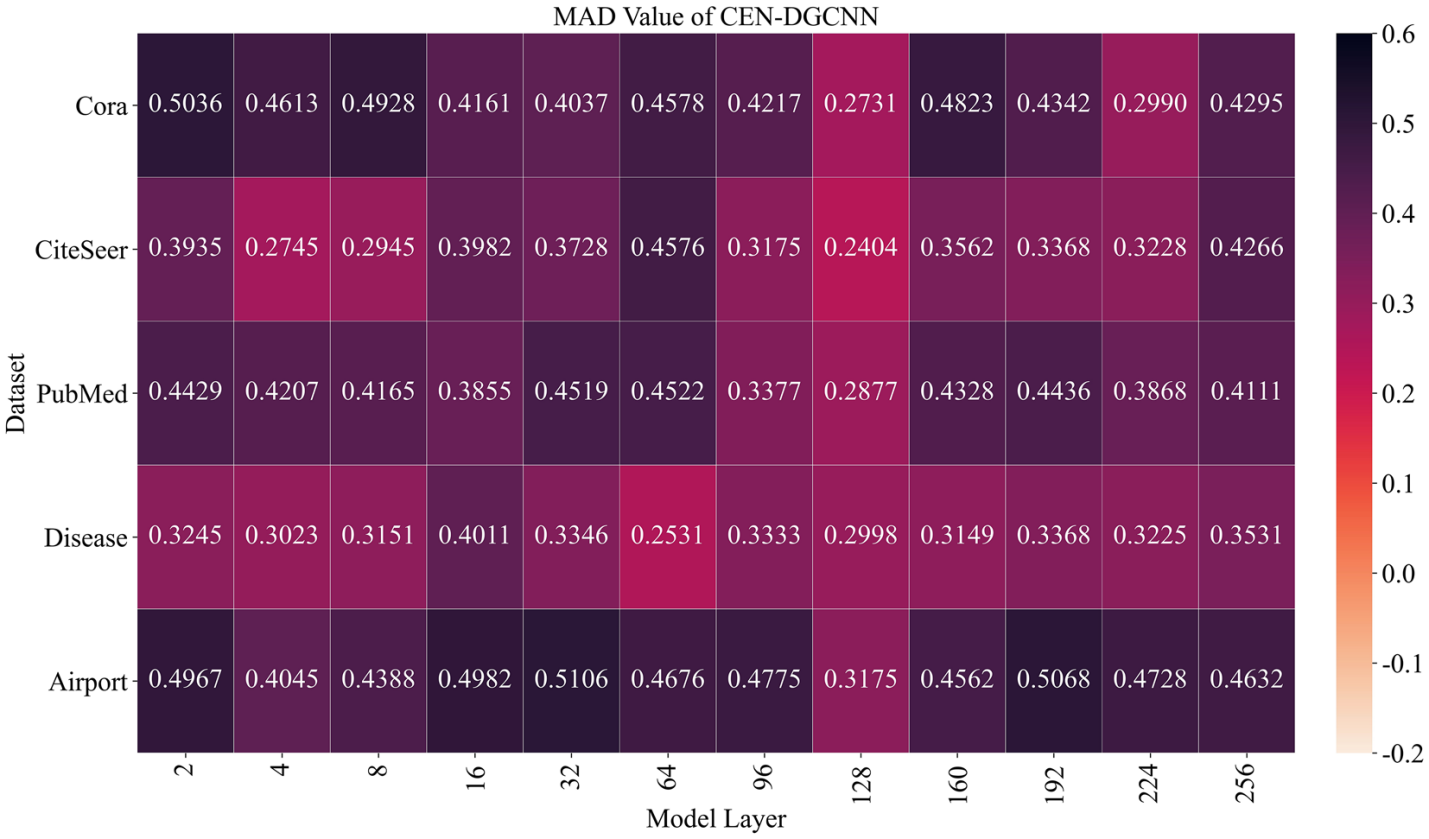
MAD values of different layers of CEN-DGCNN on 5 datasets.

**Figure 6:**
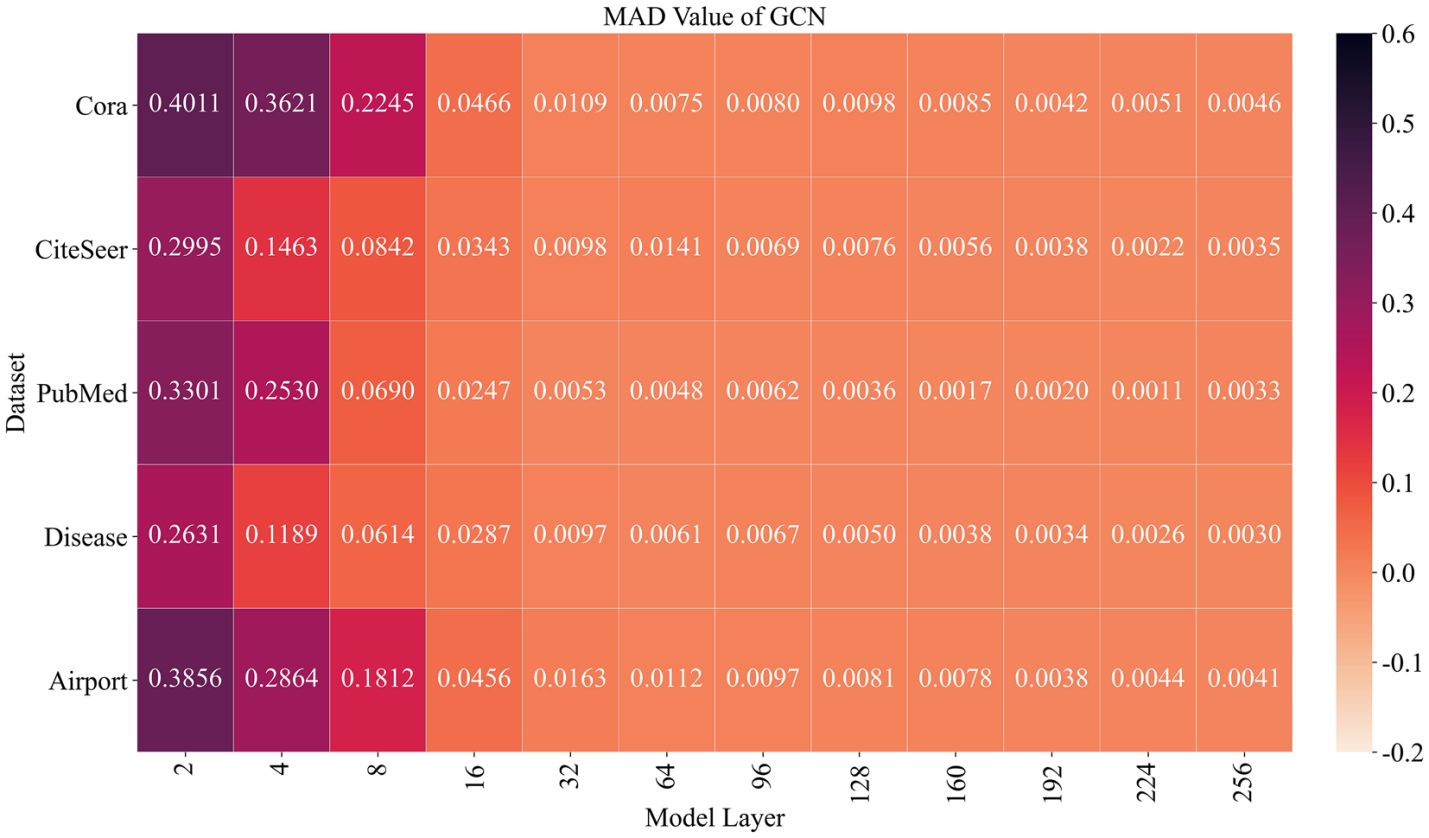
MAD values of different layers of regular GCN on 5 datasets.

Figure 5 presents the MAD values of our proposed 256-layer CEN-DGCNN on five datasets. In contrast, Fig. 6 shows the MAD values of the GCN model^2^ deepened to 256 layers on the same datasets. A higher MAD value indicates a higher degree of differentiation between nodes, i.e., a lower degree of over-smoothing. We visualized the quantized MAD values as heat maps, where darker colors indicate a smaller degree of over-smoothing, and lighter colors mean more severe node over-smoothing.

As shown in Fig. 5, the smoothness of nodes in CEN-DGCNN decreases slowly with the increase of graph convolution layers. In fact, the MAD value even increases as the number of layers deepens, indicating that the model can avoid over-smoothing. For instance, the MAD value of the 160th layer of CEN-DGCNN on the Cora dataset is higher than that of the 4th layer, and the MAD value of the 256th layer is even higher than that of the 2nd layer on the Citeseer dataset. Remarkably, the MAD value of CEN-DGCNN remains the most stable at the 64th layer. Thus, we select the 64-layer CEN-DGCNN as the default model in this study. These findings demonstrate that CEN-DGCNN has a strong ability to prevent over-smoothing of deep models. In contrast, as illustrated in Fig. 6, the conventional GCN model exhibits severe over-smoothing when the depth reaches 8 layers. As the number of layers increases, the node features become indistinguishable, making it challenging for the model to capture high-order node features and global structural information.

In order to further quantitatively analyze the over-smoothing problem, we also take the node classification accuracy as an indicator to evaluate the over-smoothing elimination. Theoretically, if the model becomes excessively smooth as the number of layers deepens, the accuracy of node classification will decline accordingly. Thus, we examine the variation in node classification accuracy of CEN-DGCNN concerning the number of layers. In Table 7, we present the node classification results for different layer models on the three citation datasets. Specifically, we compare three models: GCN, GCNII with a deep structure, EGAT with learning edge embedding, and CEN-DGCNN.

**Table 7:** Summary of classification accuracy (%) results with various depths (highest accuracy highlighted in bold).

Dataset	Method	Layers					
		2	4	8	16	32	64
Cora	GCN	**81.1**	80.4	69.5	64.9	60.3	28.7
	GCNII	80.2	82.3	82.8	83.5	84.9	**85.3**
	EGAT	**82.2**	81.3	70.6	60.1	32.8	30.1
	CEN-DGCNN	85.2	85.6	85.9	85.6	86.3	**87.1**
Citeseer	GCN	**70.8**	67.6	30.2	18.3	25.0	20.0
	GCNII	66.1	67.9	70.6	72.0	**73.2**	73.1
	EGAT	**70.2**	68.9	57.1	32.0	27.6	22.5
	CEN-DGCNN	72.8	72.8	73.4	72.9	73.1	**75.0**
Pubmed	GCN	**79.0**	76.5	61.2	40.9	22.4	35.3
	GCNII	77.7	78.2	78.8	**80.3**	79.8	80.1
	EGAT	76.3	**77.9**	70.2	52.8	28.2	30.7
	CEN-DGCNN	79.1	79.0	80.5	81.2	**81.9**	81.8

From Table 7, we observe that the performance of GCN and EGAT, which do not address the issue of over-smoothing, gradually diminishes as the model depth increases, particularly evident in the Citeseer dataset, where there is a significant performance drop when the model exceeds 8 layers. However, GCNII and CEN-DGCNN, constructed with deep model architectures, do not suffer from performance degradation with increasing model depth. On the contrary, they achieve optimal results when the number of layers is deeper. This outcome demonstrates that CEN-DGCNN effectively addresses the problem of performance degradation associated with excessively deep layers in GNNs. Additionally, CEN-DGCNN outperforms GCNII, indicating that our proposed multi-dimensional edge embedding learning method contributes to enhancing the model’s performance.

#### Qualitative analysis

**Figure 7:**
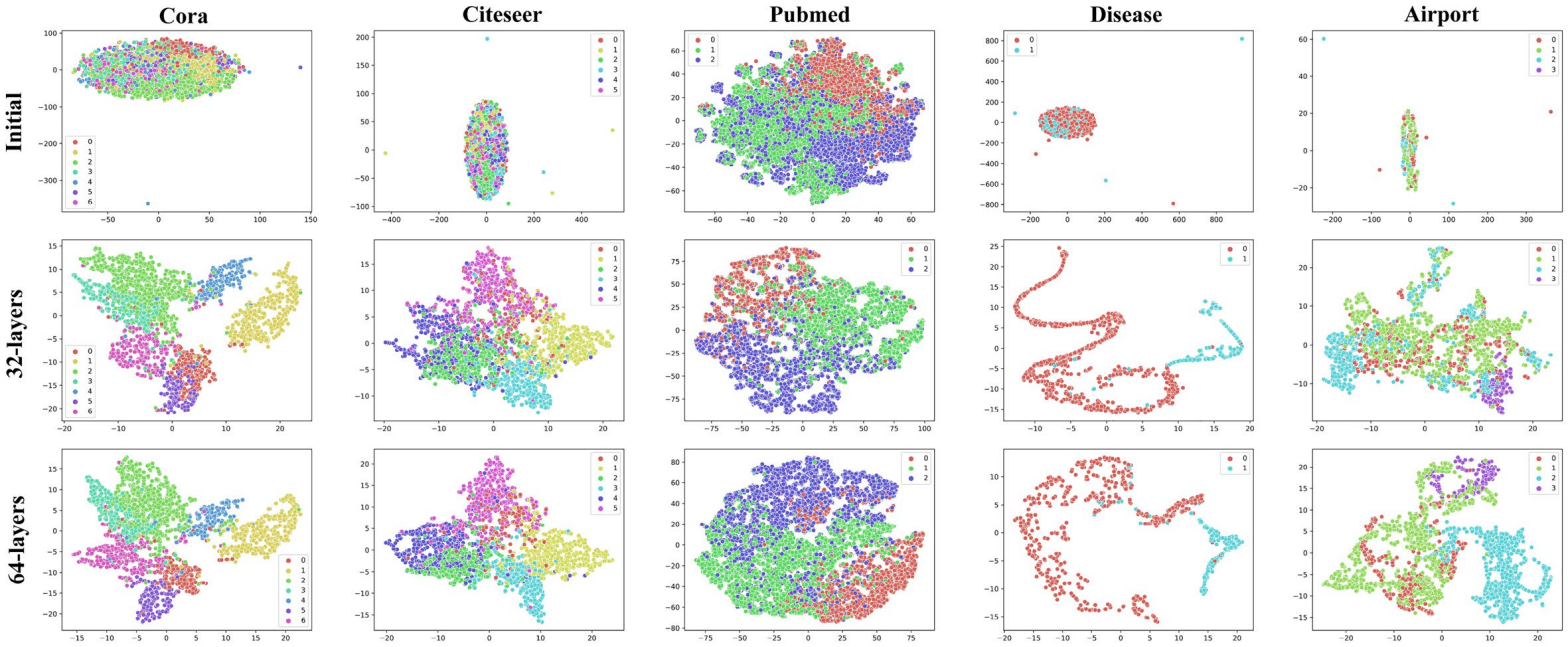
t-SNE Visualization of node representations learned by CEN-DGCNN.

The objective of the node classification task is to learn distinct node features using a GNN model. Each type of node should have similar features while being different from other types of nodes. If the classification performance is good, the nodes of the same kind will be clustered together in space, and different kinds of nodes will be highly differentiated in space. To visualize the high-order node features learned by CEN-DGCNN in two-dimensional space, we utilize the t-SNE algorithm to reduce the node features from high-dimensional to two-dimensional, and examine them in two-dimensional space. As shown in Fig. 7, the five columns from left to right represent the node feature distributions of the Cora, Citeseer, Pubmed, Disease, and Airport datasets, respectively. From top to bottom, the three rows represent the initial node features, the 32nd layer output node features of CEN-DGCNN, and the 64th layer output node features of CEN-DGCNN, respectively.

The results from Fig. 7 demonstrate that the initial node features of the five datasets are highly entangled, making it difficult to distinguish between different types of nodes and causing the same category of nodes to be randomly distributed in space. However, after the initial node features are processed by the 32-layer CEN-DGCNN, all types of nodes become clustered and distinguishable in space. After further iteration by the 64-layer CEN-DGCNN, a clear boundary appears between different types of nodes in all five datasets, and the same types of nodes are tightly clustered together. Thus, our qualitative analysis suggests that the deep structure of CEN-DGCNN effectively mitigates the node over-smoothing problem and achieves remarkable node classification performance.

### Ablation experiments

In this section, we will carry out ablation experiments from the two perspectives of our proposed multidimensional edge feature encoding method and a new message passing framework to prove the effectiveness of the above multidimensional edge feature encoding method and the new framework. Through the experiments in this section, we will prove the following three points: (1) Our proposed multi-dimensional edge feature encoding method can significantly improve node classification accuracy, achieving up to 55.19% improvement in the five datasets with missing edge features, even without the use of our novel messaging framework. (2) Our novel message passing framework is robust to deep graph convolutional neural networks encoded with low-dimensional edge features. In the case of low-dimensional edge feature encoding, using a deep graph convolutional neural network model based on a new message passing framework can improve the node classification task by about 27.04–53.7% accuracy. (3) Using the new message passing framework and multi-dimensional edge feature encoding method at the same time will achieve better classification results.

#### Different edge feature encoding methods

In this section, we aim to validate the effectiveness of the proposed multi-dimensional edge feature encoding method for the missing edge feature network. The conventional GNNs can only learn node features and not edge features, whereas our proposed CEN-DGCNN can update multi-dimensional edge feature vectors across layers, making it possible to learn various features of edges such as categories, attributes, directions, and connection strengths automatically. However, many datasets have fewer edge features, such as the edges of the Citation Network dataset that only contain direction features. To address this issue, we propose a multi-dimensional edge feature encoding method as shown in Eq. (16) for the edge directionality of directed graph datasets.

To verify the effectiveness of the proposed multi-dimensional edge feature encoding method, we will compare its performance with that of various low-dimensional edge feature representations. We construct these low-dimensional edge feature representations in three specific ways: (1) Single-channel edge feature construction method that only includes one-way edge, namely $\left[{E}_{ij}\right]$ and $\left[{E}_{ji}\right]$. (2) Single-channel edge feature construction method regarded as undirected graph, namely $\left[{E}_{ij}+{E}_{ji}\right]$. (3) Dual-channel edge feature construction method with bi-directional edges, namely $\left[{E}_{ij}, {E}_{ji}\right]$. The node classification accuracies of our proposed multi-dimensional edge feature construction method and the above low-dimensional edge construction method on the five datasets are shown in Table 8.

**Table 8:** Accuracy of node classification under different edge feature encoding methods (highest accuracy highlighted in bold).

Edge encoding	Cora (%)	Citeseer (%)	Pubmed (%)	Disease (%)	Airport (%)
$\left[{E}_{ij}\right]$	59.71	67.33	69.65	60.32	70.12
$\left[{E}_{ji}\right]$	60.12	68.79	69.11	61.61	71.32
$\left[{E}_{ij}+{E}_{ji}\right]$	84.74	70.81	70.31	71.34	80.92
$\left[{E}_{ij}, {E}_{ji}\right]$	86.21	74.13	81.12	73.11	83.79
$\left[{E}_{ij}+{E}_{ji}, {E}_{ij}, {E}_{ji}\right]$	**87.13**	**75.01**	**81.82**	**73.52**	**85.85**

**Figure 8:**
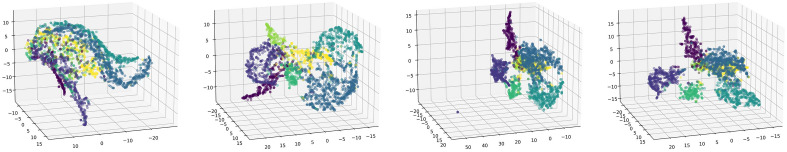
Node feature visualization results of CEN-DGCNN with various edge feature encoding methods in the Cora dataset.

Table 8 demonstrates that our proposed multi-dimensional edge construction method outperforms all low-dimensional edge construction methods in node classification tasks. Figure 8 shows the t-SNE visualization results of 64-layer CEN-DGCNN using four edge construction methods to classify nodes in the Cora dataset, the edge construction methods from left to right are $\left[{E}_{ij}\right]$, $\left[{E}_{ij}+{E}_{ji}\right]$, $\left[{E}_{ij}, {E}_{ji}\right]$, $\left[{E}_{ij}+{E}_{ji}, {E}_{ij}, {E}_{ji}\right]$. The t-SNE algorithm reduces the high-dimensional node features output by the CEN-DGCNN to a three-dimensional space. As seen in Fig. 8, the seven types of nodes outputted by the low-dimensional edge construction method are closely clustered and confused in space. However, the node features obtained by our proposed edge construction method are clearly classified in space, resulting in the best clustering effect for similar nodes. This demonstrates that the multi-channel edge feature coding method has a better classification effect than the low-channel edge feature coding method.

We analyze that the effectiveness of the multi-dimensional edge feature construction method in enhancing model performance is attributed to utilizing edge feature matrices of different channels as filters to aggregate node features. This facilitates nodes in acquiring more comprehensive information, this is equivalent to aggregating node features from diverse edge dimensions, and ultimately combining the node features aggregated across these different dimensions.

Taking the example of the three-dimensional edge feature representation method based on edge directionality, nodes engage in message passing and aggregation from three edge directions. The edge features are learned and updated at each layer, and eventually, node features based on multiple edge directions are combined. The node features obtained through this approach contain significantly richer information compared to the conventional method of filtering with an invariant adjacency matrix for each layer. Consequently, the utilization of multi-dimensional edge features empowers the model to better capture the intricate relationships among nodes, leading to improved model performance.

#### The effectiveness of the novel message passing framework

In order to capture long-range dependencies between nodes and obtain more refined high-order node features and non-local structural features, we propose a new message passing framework shown in Eq. (6). To demonstrate its effectiveness, we conduct two sets of model comparison experiments. The first set of models includes CEN-DGCNN with four different edge construction methods, but using the traditional ***MPNN*** framework instead of our novel message passing framework. The second set of models also includes CEN-DGCNNs with four different edge construction methods, but uses our proposed novel message passing framework (***Ours***). The node classification results of the eight CEN-DGCNNs and their variant models are shown in Table 9. From the experimental data, it can be seen that our proposed multi-dimensional edge feature construction method combined with the novel message passing framework achieves the best node classification results. Moreover, compared with the same edge feature construction method, the node classification performance of the low-dimensional edge feature construction model is significantly improved after using the novel message passing framework. These results suggest that the novel message passing framework is robust to deep graph neural networks using low-dimensional edge feature construction methods.

**Table 9:** Node classification accuracy with different message passing frameworks (highest accuracy highlighted in bold).

Model	Cora (%)	Citeseer (%)	Pubmed (%)	Disease (%)	Airport (%)
$\left[{E}_{ij}\right]$+ ***MPNN***	31.16	22.78	29.83	23.03	31.23
$\left[{E}_{ij}+{E}_{ji}\right]$+ ***MPNN***	31.51	25.18	30.54	25.34	30.56
$\left[{E}_{ij}, {E}_{ji}\right]$+ ***MPNN***	84.37	70.22	76.78	69.27	81.78
$\left[{E}_{ij}+{E}_{ji}, {E}_{ij}, {E}_{ji}\right]$+***MPNN***	86.54	73.85	80.26	72.34	84.07
$\left[{E}_{ij}\right]$+ ***Ours***	59.71	67.33	69.65	60.32	70.12
$\left[{E}_{ij}+{E}_{ji}\right]$+ ***Ours***	84.75	70.81	70.31	71.34	80.92
$\left[{E}_{ij}, {E}_{ji}\right]$+ ***Ours***	86.21	74.13	81.12	73.11	83.79
$\left[{E}_{ij}+{E}_{ji}, {E}_{ij}, {E}_{ji}\right]$+ ***Ours***	**87.13**	**75.01**	**81.82**	**73.52**	**85.85**

**Figure 9:**
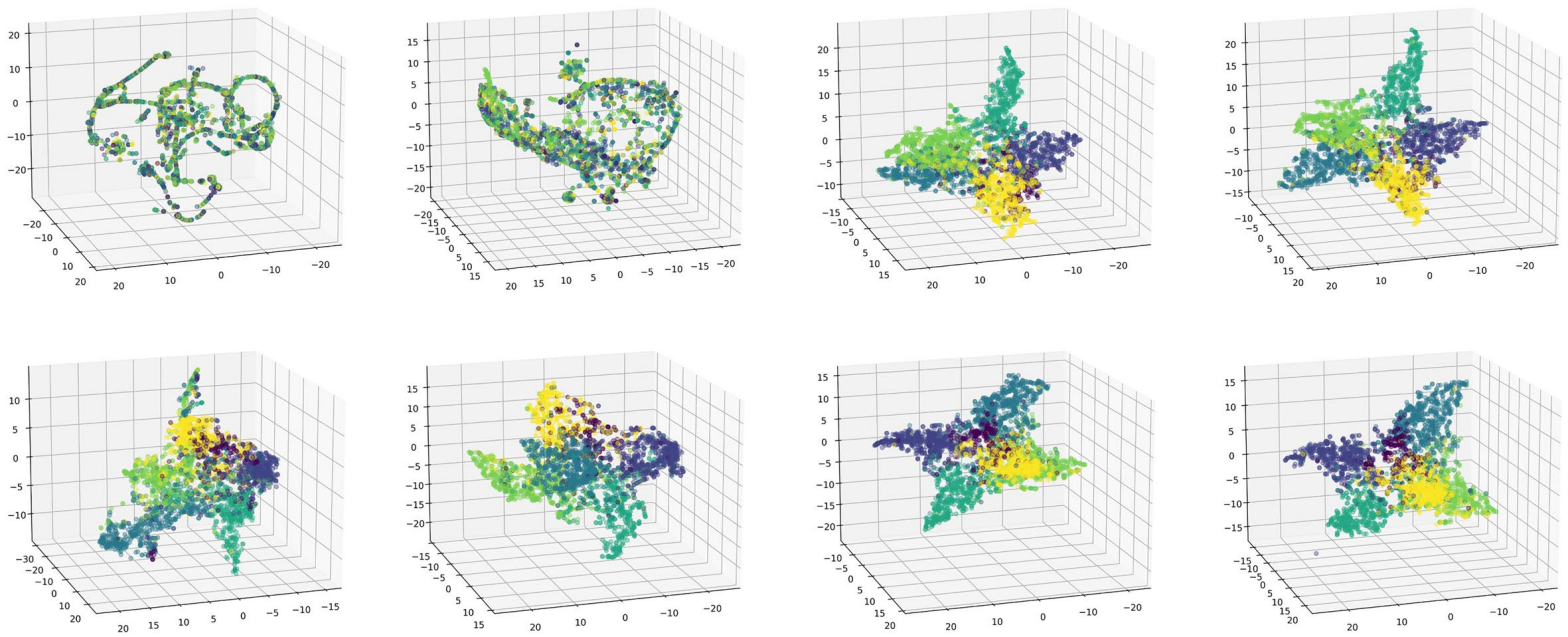
Node feature visualization results of CEN-DGCNN with different message passing frameworks and edge feature construction methods in the Citeseer dataset.

Figure 9 shows the t-SNE visualization results of all models in Table 9 for node classification on the Citeseer dataset. The upper and lower lines represent the message passing framework using MPNN and our novel messaging framework, respectively. Each column from left to right represents the edge feature encoding method of $\left[{E}_{ij}\right]$, $\left[{E}_{ij}+{E}_{ji}\right]$, $\left[{E}_{ij}, {E}_{ji}\right]$, $\left[{E}_{ij}+{E}_{ji}, {E}_{ij}, {E}_{ji}\right]$. By comparing the first two columns, it can be seen that the novel messaging framework performs strongly in the case of low-dimensional edge construction. The node features of the first two columns in the upper row are almost indistinguishable in three-dimensional space, while the first two columns in the lower row achieve better node classification results. As seen in the last column, the best node classification results are achieved when using both the novel message passing framework and the multi-dimensional edge feature construction method.

### Model analysis

In this section, we will analyze the model complexity of CEN-DGCNN to explore the effects of simultaneously learning node embeddings and edge embeddings on model complexity. Additionally, we will investigate the sensitivity of the hyperparameter “$\lambda$” in Eq. (10) on model performance. Moreover, we will compare the attention distributions of several representative models to highlight the advantages of CEN-DGCNN.

#### Model complexity analysis

We analyze the complexity of CEN-DGCNN to highlight its advantages in performance. We discuss in detail the memory complexity, parameter complexity and time complexity of full-batch GNN, GraphSAGE, ClusterGCN, FastGCN and RevGNN models which can reach 1000 layers, and compare the complexity of our proposed CEN-DGCNN with these models. In Table 10, we summarize the theoretical complexity of all models, where $L$ represents the number of model layers, $N$ represents the number of nodes, $B$ represents the batch size of nodes, $D$ represents the number of hidden channels, $K$ represents the number of neighbor samples for each node, ${\Vert A\Vert }_{0}$ denotes the sparsity of the graph, specifically the number of non-zero elements in the graph's adjacency matrix $A$, and $P$ represents the dimension of multi-dimensional edge feature encoding. It is noteworthy that during our experiments, the use of double random matrix calculations for denoising the edge feature matrix may, in fact, adversely affect the model's operational efficiency. In scenarios where the hardware platform’s performance is constrained, we recommend relocating the double random matrix denoising operation to the data preprocessing stage. This adjustment can help alleviate resource overhead during model execution. Given the negligible memory footprint occupied by model parameters, our primary focus in this analysis centers on the memory complexity required to store intermediate node features.

**Table 10:** Comparison of complexities.

Method	Memory	Params	Time
Full-batch GNN	$O(LND)$	$O(L{D}^{2})$	$O(L{\Vert A\Vert }_{0}D+LN{D}^{2})$
GraphSAGE^54^	$O({K}^{L}BD)$	$O(L{D}^{2})$	$O({K}^{L}N{D}^{2})$
ClusterGCN^59^	$O(LBD)$	$O(L{D}^{2})$	$O(L{\Vert A\Vert }_{0}D+LN{D}^{2})$
FastGCN^63^	$O(LKBD)$	$O(L{D}^{2})$	$O(KLN{D}^{2})$
RevGNN^58^	$O(ND)$	$O(L{D}^{2})$	$O(L{\Vert A\Vert }_{0}D+LN{D}^{2})$
CEN-DGCNN	$O(PLND)$	$O(PL{D}^{2})$	$O(PL{\Vert A\Vert }_{0}D+PLN{D}^{2})$
CEN-DGCNN + Subgraph sampling	$O(PLBD)$	$O(PL{D}^{2})$	$O(PL{\Vert A\Vert }_{0}D+PLN{D}^{2})$

It is evident from the data presented in Table 10 that the memory consumption of RevGNN is independent of depth. Consequently, RevGNN can construct deeper models within the same memory space. However, as demonstrated by the experimental results in Section “Results and analysis”, when model depth is held constant, CEN-DGCNN achieves superior performance to RevGNN while utilizing only 6% of the number of channels employed by RevGNN. This observation holds great significance in terms of reducing parameter complexity and time complexity. As revealed by the data in Table 10, the $D$ parameter within the model's parameter complexity and time complexity exhibits a quadratic growth pattern. An increase in the number of hidden channels, $D$, results in an exponential rise in the number of parameters and training time. Conversely, the $P$ term introduced by CEN-DGCNN contributes linearly to the increment in parameter complexity and time complexity. What distinguishes CEN-DGCNN is its ability to exponentially reduce parameter complexity and time complexity by decreasing the number of channels, which outpaces the linear complexity increase brought about by the new $P$ term. Our approach can also be integrated with mini-batch sampling techniques to further mitigate memory complexity in terms of the number of nodes.

**Figure 10:**
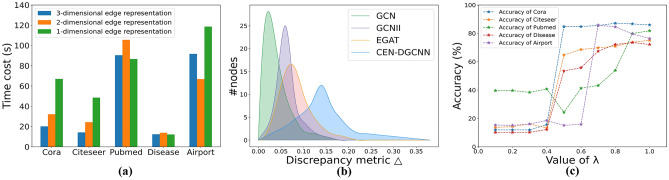
CEN-DGCNN Model Analysis. (**a**) The running time of the CEN-DGCNN model under different-dimensional edge feature constructions. (**b**) Attention weight distribution on the Citeseer dataset. (**c**) The performance of CEN-DGCNN is influenced by the value of the hyperparameter $\lambda$.

However, in practice, learning multi-dimensional edge embeddings may accelerate the model’s convergence speed, leading to shorter learning times compared to low-dimensional edge embeddings. Figure 10a illustrates a comparison of the runtime of the CEN-DGCNN model using edge features constructed in different dimensions. It can be observed that in the case of 3-dimensional edge feature construction, the runtime is not necessarily longer than that in the case of low-dimensional edge feature construction.

#### Attention distribution

To demonstrate that CEN-DGCNN achieves higher attention scores through the learning of edge features, we will analyze the attention scores learned by four models, namely GCN, GCNII, EGAT, and CEN-DGCNN. First, we define the discrepancy measure on the attention matrix $A$ of nodes ${v}_{i}$ as ${\Delta }_{i}=\frac{\Vert {A}_{\left[i,:\right]}-{U}_{i}\Vert }{degree({v}_{i})}$ ^65^, where ${U}_{i}$ represents the uniform distribution score of nodes ${v}_{i}$. ${\Delta }_{i}$ is used to quantify the deviation of the learned attention from the uninformative uniform distribution. A larger ${\Delta }_{i}$ indicates that the learned attention scores are more meaningful. Figure 10b illustrates the distribution of discrepancy metrics for the attention matrices learned by the four models on the Citeseer dataset. It can be observed that the attention scores learned by CEN-DGCNN exhibit larger variance. This indicates that CEN-DGCNN outperforms the other models, as it better distinguishes important nodes and learns the corresponding attention scores more effectively.

#### Parameter sensitivity analysis

For ensuring a fair comparison of our experiments, we have already introduced the relevant experimental parameter settings in Section “Model settings”. In this section, we will conduct a sensitivity test for the significant adaptive decay parameter ${\delta }_{l}$ (Eq. 10) in the CEN-DGCNN model. Figure 10c illustrates the node classification accuracy of the 64-layer CEN-DGCNN concerning the hyperparameter $\lambda$ in ${\delta }_{l}$. By adjusting the value of $\lambda$, we can control the extent of information decay in the model during the feature transformation stage. From the Fig. 10c, it can be observed that when the value of $\lambda$ is below 0.5, the model’s performance experiences a significant decline. Different datasets have corresponding optimal $\lambda$ values, with the optimal values typically ranging between 0.5 and 1.

## Conclusion

In this paper, we first introduce a multi-dimensional edge feature representation method that overcomes the limitations of conventional GNNs, which can only use binary edge representation and one-dimensional edge feature representation. Our method enables the update and learning of multi-dimensional edge features across layers in CEN-DGCNN, providing a basis for downstream tasks. In each graph convolution layer, the multi-dimensional edge feature matrix can also be used as a multi-channel filter to filter node features. By updating the multi-dimensional edge features and node features synchronously, our model reduces complexity and improves computational efficiency. Additionally, we propose a novel message passing framework to obtain more refined high-order features of nodes, capturing remote dependencies between nodes and global structure features. CEN-DGCNN, based on this framework, achieves a very deep network structure, and eliminates node over-smoothing problem, thus performing better than the shallow structure. We analyze the node smoothness of CEN-DGCNN quantitatively and qualitatively in each layer, proving that it can perfectly solve the problem of node over-smoothing. Finally, we demonstrate the superior performance of CEN-DGCNN compared to a large number of baseline GNN models. We also prove the efficacy of the multi-dimensional edge feature construction method and the new message passing framework through ablation experiments. We aim to apply CEN-DGCNN to more areas and tasks in the future and continue to improve our model.

## Data Availability

All datasets used in this paper are available in the GitHub repository: https://github.com/ytchx1999/GraphSAGE-Cora-Citeseer-Pubmed/tree/main/data; https://github.com/HazyResearch/hgcn/tree/master/data.
